# Multi-scaled transcriptomics of chronically inflamed nasal epithelium reveals immune-epithelial dynamics and tissue remodeling in nasal polyp formation

**DOI:** 10.1016/j.immuni.2025.08.009

**Published:** 2025-09-12

**Authors:** Guanrui Liao, Tsuguhisa Nakayama, Bokai Zhu, Ivan T. Lee, Jason Yeung, Yao Yu Yeo, Yuzhou Chang, Cankun Wang, Steven Chun-Kang Liao, Dingani Nkosi, Axel Renteria, Dawn T. Bravo, Jonathan B. Overdevest, Carol H. Yan, David Zarabanda, Philip A. Gall, Sachi S. Dholakia, Nicole A. Borchard, Angela Yang, Dayoung Kim, Zara M. Patel, Peter H. Hwang, Dhananjay Wagh, John Coller, Katie M. Phillips, Michael T. Chang, Matt Lechner, Zihai Li, Te-Huei Yeh, Garry Nolan, Maria Serena Longhi, Vassiliki Boussiotis, Dan H. Barouch, Qin Ma, Jayakar V. Nayak, Sizun Jiang

**Affiliations:** 1 Center for Virology and Vaccine Research, Beth Israel Deaconess Medical Center, Boston, MA 02115, USA; 2 Department of Otolaryngology-Head and Neck Surgery, Stanford University School of Medicine, Stanford, CA 94305, USA; 3 Department of Pathology, Stanford University School of Medicine, Stanford, CA 94305, USA; 4 Department of Biomedical Informatics, The Ohio State University, Columbus, OH 43210, USA; 5 Pelotonia Institute for Immuno-Oncology, The James Comprehensive Cancer Center, The Ohio State University, Columbus, OH 43210, USA; 6 Department of Otolaryngology, National Taiwan University Hospital, Yunlin Branch, Yunlin County, Douliu 640203, Taiwan; 7 Department of Pathology, Brigham and Women’s Hospital, Harvard Medical School, Boston, MA 02115, USA; 8 Department of Otolaryngology-Head and Neck Surgery, Columbia University School of Medicine, New York, NY 10032, USA; 9 Division of Otolaryngology-Head and Neck Surgery, Department of Surgery, University of California, San Diego School of Medicine, University of California, San Diego, La Jolla, CA 92093, USA; 10 Department of Genetics, Stanford University School of Medicine, Stanford, CA 94305, USA; 11 Department of Otolaryngology-Head and Neck Surgery, University of Cincinnati School of Medicine, Cincinnati, OH 45221, USA; 12 UCL Head and Neck Academic Center, Division of Surgery and Interventional Science, University College London, London WC1E 6BT, UK; 13 Department of Otolaryngology, National Taiwan University Hospital Hsin-Chu Branch, Biomedical Park Hospital, Hsinchu 302058, Taiwan; 14 Department of Anesthesia, Critical Care & Pain Medicine, Beth Israel Deaconess Medical Center, Harvard Medical School, Boston, MA 02115, USA; 15 Department of Hematology-Oncology, Beth Israel Deaconess Medical Center, Harvard Medical School, Boston, MA 02115, USA; 16 Department of Otolaryngology-Head and Neck Surgery, Veterans Affairs Palo Alto Health Care System, Palo Alto, CA 94304, USA; 17 Department of Pathology, Dana Farber Cancer Institute, Boston, MA 02115, USA; 18 The Broad Institute of Harvard and MIT, Cambridge, MA 02142, USA; 19 Present address: Center of Hepato-Pancreato-Biliary Surgery, The First Affiliated Hospital of Sun Yat-sen University, Guangzhou, Guangdong 510080, China; 20 Present address: Department of Otorhinolaryngology-Head and Neck Surgery, Dokkyo Medical University, Tochigi 321-0293, Japan; 21 Present address: Ragon Institute of MGH, MIT, and Harvard, Cambridge, MA 02139, USA; 22 Present address: Division of Allergy and Immunology, Boston Children’s Hospital, Harvard Medical School, Boston, MA 02115, USA; 23 These authors contributed equally; 24 Lead contact

## Abstract

Chronic rhinosinusitis (CRS) is a common chronic inflammatory disease of the sinonasal cavity affecting millions worldwide. Its complex pathophysiology remains poorly understood, with emerging evidence implicating interactions between diverse immune and epithelial cells in disease progression. We applied single-cell RNA sequencing (scRNA-seq) and spatial transcriptomics to both dissociated and intact human tissues from individuals with CRS with and without nasal polyps and compared them with controls. We revealed mechanisms of macrophage-eosinophil recruitment, CD4^+^ and CD8^+^ T cell dysregulation, and mast cell enrichment. We identified key immune-epithelial interactions in tissue remodeling, particularly involving basal progenitor and tuft cells. A distinct basal cell trajectory was implicated in nasal polyp formation. Orthogonal validation with spatial transcriptomics from >100 individuals with CRS revealed conserved tissue remodeling features. Our study provides insights into CRS pathophysiology, highlighting immune-epithelial interactions as potential therapeutic targets in chronic inflammation, also serving as a resource for dissecting immune disease mechanisms.

## In brief

Chronic rhinosinusitis (CRS) is driven by poorly understood immune-epithelial crosstalk. Liao et al. present a single-cell and spatial transcriptomic atlas of CRS, revealing how immune and epithelial cells interact to drive tissue remodeling and inflammation. This resource provides insight into disease mechanisms and offers a valuable framework for studying chronic inflammatory disorders of the airway.

## Graphical Abstract

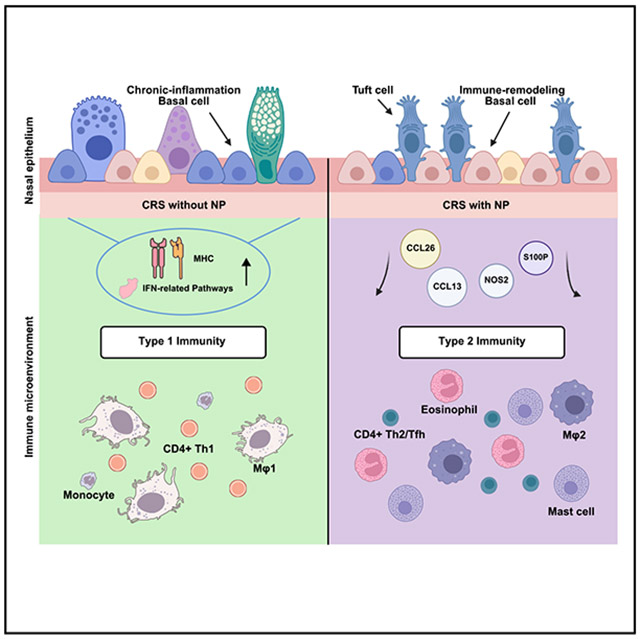

## INTRODUCTION

Chronic rhinosinusitis (CRS) is a heterogeneous inflammatory disease of the nasal and sinonasal cavities. Its global prevalence is estimated at 12%,^1,2^ with symptom severity comparable to heart disease and chronic back pain.^1^ CRS is classified into two subtypes based on the presence or absence of nasal polyps (NPs): CRS with nasal polyps (CRSwNP) and CRS without nasal polyps (CRSsNP). CRSsNP typically accounts for 75%–80% of cases vs. 20%–25% for CRSwNP,^3^ although this varies regionally. CRSwNP is associated with increased disease burden due to eosinophil-rich polyposis and inflammation, leading to recalcitrant symptoms such as headaches, olfactory loss, and recurrent sinusitis. While both innate and T helper 2 (Th2) cell-acquired immunity are involved, how immune (IMM) and epithelial (EPI) interactions drive NP formation remains a mystery.

IMM-EPI crosstalk plays a critical role in the pathogenesis of many diseases as well as regeneration, including CRS.^4-6^ The nasal epithelium acts as a responsive physical barrier against environmental challenges from pathogens, pollutants, and allergens. IMM cells, including T cells, B cells, dendritic cells (DCs), and mast cells (MCs), recognize insults and coordinate immunity. The interaction between EPI cells and IMM cells is complex and dynamic, involving a range of autocrine and paracrine signaling molecules, cytokines, and chemokines. For example, EPI cells secrete cytokines that recruit leukocytes via innate and acquired immunity. In turn, IMM cells can also yield cytokines that stimulate or blunt EPI cell function, thus orchestrating other IMM pathways. This bidirectional interplay remains a major barrier to understanding CRS tissue remodeling.

T cells, MCs, and other IMM players contribute substantially to CRS pathogenesis through diverse signaling and effector functions. CD4 T cells differentiate into subsets like Th2 cells, which produce the cytokines interleukin (IL)-4, IL-5, and IL-13 to recruit eosinophils and MCs: key mediators in CRSwNP.^7^ CD8^+^ T cells eliminate infected or damaged cells, although their roles in CRS remain under characterized. MCs further propagate inflammation via histamine, prostaglandins, and leukotrienes^8^; their infiltration and activation are elevated in CRSwNP with further ties to eosinophilic inflammation.^9^

Basal EPI cells also play a central role in CRS tissue remodeling. These progenitor cells reside along the basement membrane and differentiate into ciliated and goblet cells. Hyperplasia of basal cells is a hallmark of CRSwNP,^10,11^ but its pathological relevance remains unclear. Environmental stress may prompt basal cell differentiation that primes IMM-EPI crosstalk.^12,13^ Our prior work showed that type 2 inflammation in macrophages and EPI subsets contributes to the IMM landscape in CRSwNP.^14^

To further dissect these interactions, we here applied single-cell RNA sequencing (scRNA-seq) to a discovery CRS cohort and validated our findings using spatial transcriptomics across two large multi-center cohorts from North America and Asia. This integrative approach illuminates IMM-EPI crosstalk such as a basal cell state central to IMM cell recruitment, NP formation, and tissue remodeling in CRSwNP, thus offering a valuable resource for broader studies in chronic inflammation and remodeling diseases.

## RESULTS

### Single-cell transcriptomic analysis of the CRS microenvironment

**Figure 1.**
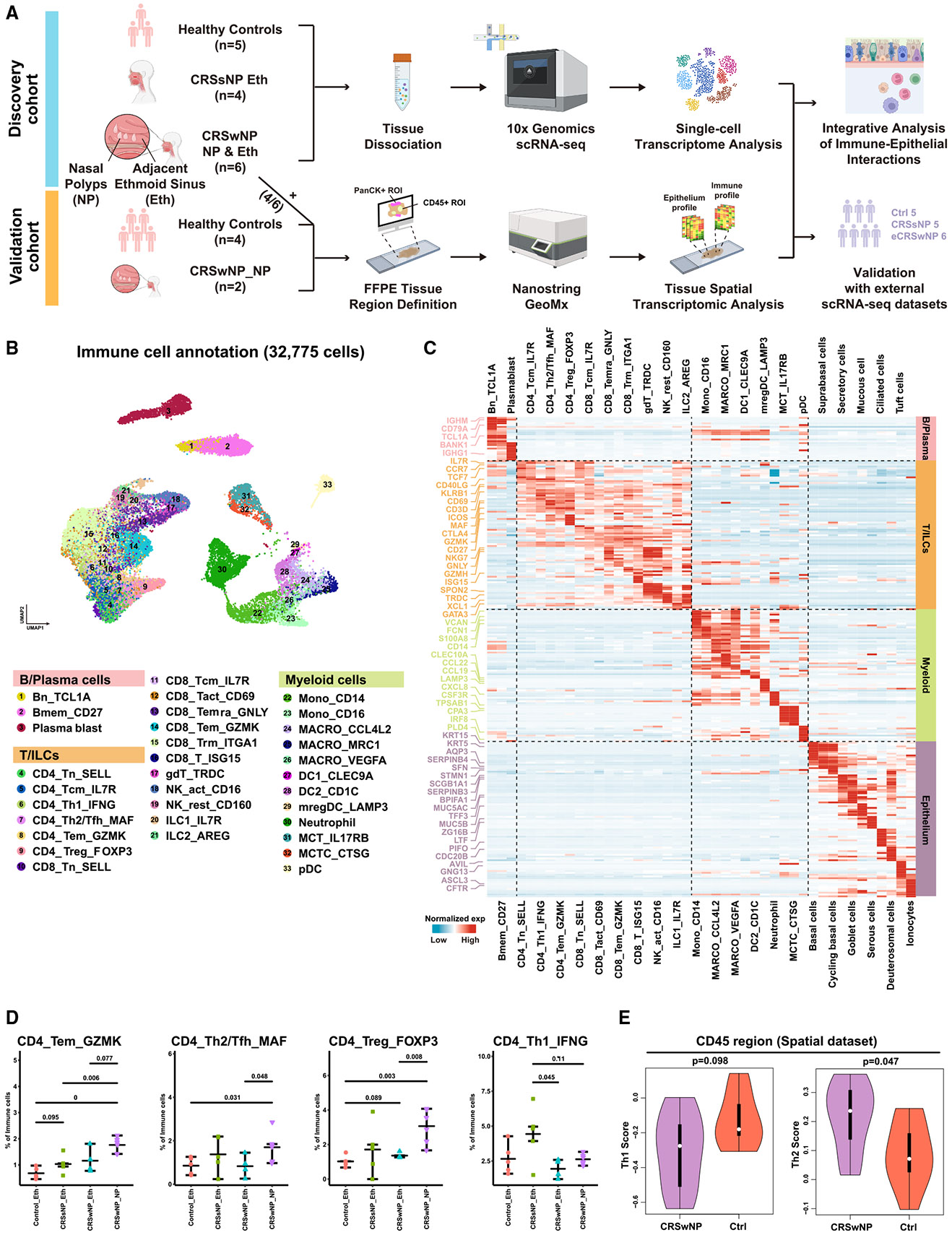
In-depth analysis of the IMM and EPI cellular landscape in CRS using multi-scaled transcriptomics (A) Schematic of the two-cohort design: a discovery cohort (scRNA-seq) and a validation cohort (spatial transcriptomics). Discovery included healthy controls (*n* = 5), CRSsNP ethmoid (*n* = 4), and CRSwNP polyp + adjacent ethmoid (*n* = 6); validation included healthy controls (*n* = 4) and CRSwNP polyps (*n* = 2). eCRSwNP, eosinophilic CRSwNP. (B) UMAP visualization of 32,775 IMM cells, grouped into 3 lineages (B/plasma, T/ILCs, MLC) and 33 subtypes, colored by cell type. (C) Heatmap depicting normalized expression patterns of signature genes across the identified IMM and EPI cell populations, with distinct gene modules highlighted for B/plasma cells, T/ILCs, MLCs, and EPI cells. (D) Frequency comparison of key CD4^+^ T cell subtypes between control and CRS samples. Statistical significance was determined using two-sided Wilcoxon tests. Bars represent the data range, with the lines indicating the median. (E) Violin plots comparing Th1 and Th2 signature scores between CRSwNP and control samples within CD45^+^ regions from spatial transcriptomics data, demonstrating enhanced Th2 and reduced Th1 signatures in CRSwNP. Boxes indicate interquartile range (25th–75th), with the dot indicating the median. See also Figure S1.

We utilized single-cell transcriptomics for an in-depth analysis of the CRS EPI and IMM landscape on an initial discovery cohort of rigorously selected individuals (*n* = 5 healthy controls, *n* = 5 CRSsNP, and *n* = 6 CRSwNP for both the NP and adjacent non-polyp ethmoid sinus mucosa; see STAR Methods and Table S1) (Figures 1A and S1A). We first identified the major IMM cell types within the IMM microenvironment (Figure 1B), as depicted in the uniform manifold approximation and projection (UMAP) plot. These IMM cells were primarily of the B, T, and myeloid lineages (32,775 cells in total). The origin of IMM cells was visualized on a UMAP plot, color-coded by tissue types and individual samples (Figure S1B). Representative marker genes for the different cellular populations across the IMM cell repertoire are depicted on the UMAP (Figure S1C) and in heatmap form (Figure 1C; Table S2).

We first performed cell frequency comparisons between samples in T cell populations: comparisons of CD4^+^ T cells and their subtypes between CRS and control samples revealed several categories represented across healthy and CRS samples (Figure 1D). We identified an enrichment of CD4^+^ T effector memory (Tem), Th2, and T regulatory (Treg) CD4^+^ subtypes in CRSwNPs and a Th1 skew in CRSsNP as previously described.^15^ We also confirmed an elevated immunosuppressive CD4^+^ T cell state within CRSwNP compared with CRSsNP—as demonstrated by a reduction of IMM cells related to a Th1 pathway—and enrichment of IMM cells skewed toward a Th2 pathway, from spatial transcriptomics (Figure 1E). Similarly, we investigated and identified leukocyte subtypes and corresponding annotations in CD8^+^ T cells in both CRS and control samples (Figure S1D). In line with our CD4^+^ T cell analysis, we also identified the enrichment of Tems in the CRSwNP samples, along with a reduction in CD8^+^ resident memory T cell phenotypes (Figure S1D). These results together support a model in which suppressor and regulatory T cells, including players involved in a type 2 IMM response, are responsible for the chronic inflammatory features of CRSwNP, compared with CRSsNP.^16^

### Macrophage cell-state adaptation in CRS NPs

**Figure 2.**
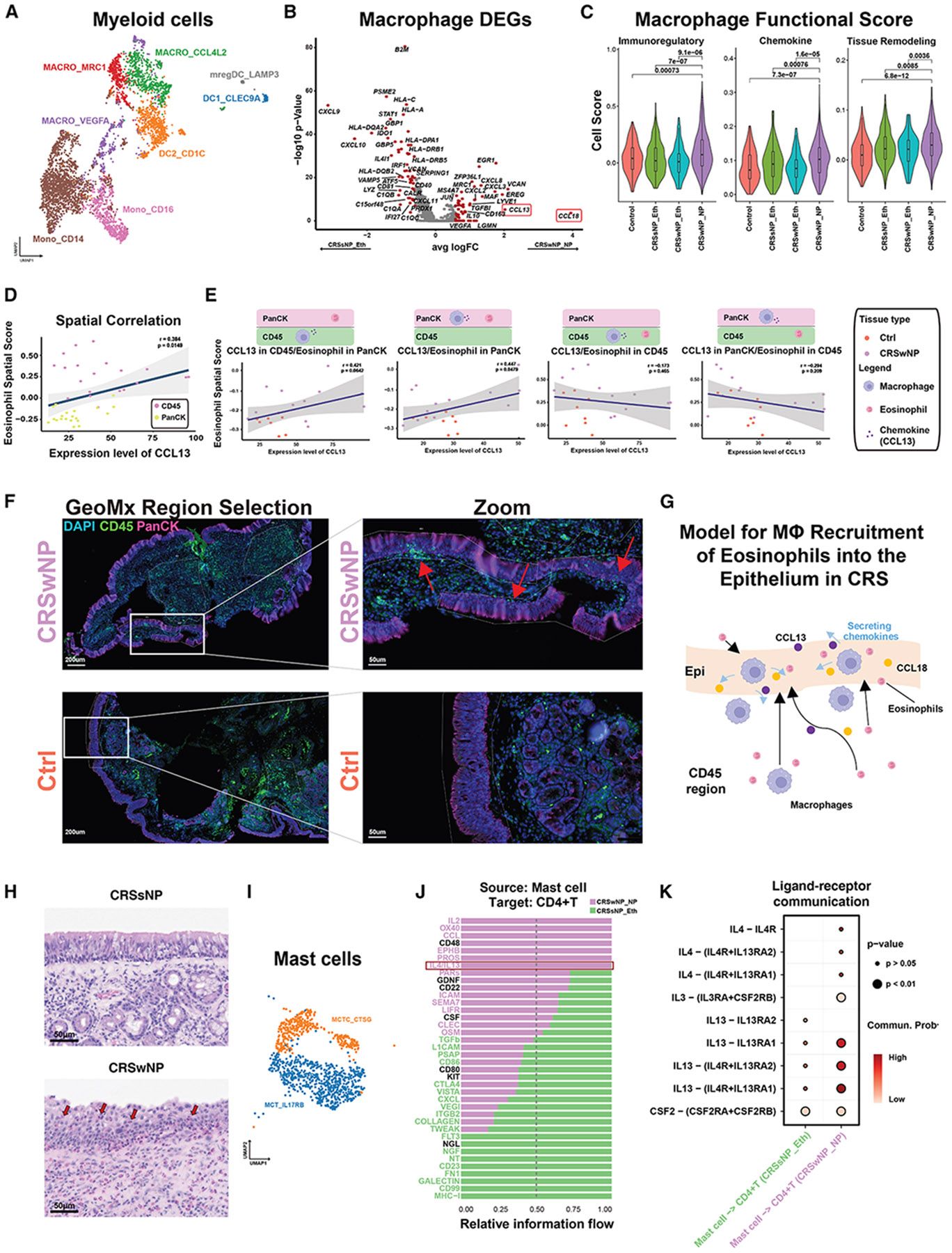
Macrophage cell-state adaptation and MC enrichment correlate with type 2 inflammation in CRSwNP (A) UMAP of MLC populations, revealing distinct macrophage subtypes (MACRO_MRC1, MACRO_CCL4L2, and MACRO_VEGFA), DCs (DC1_CLEC9A and DC2_CD1C), monocytes (Mono_CD14 and Mono_CD16), and other. (B) Volcano plot of differentially expressed macrophage genes between CRSsNP and CRSwNP. Red dots highlight genes with ∣log_2_FC∣ > 1.5, including *CCL13* and *CCL18*. (C) Violin plots show higher immunoregulatory polarization scores in CRSwNP polyp macrophages vs. control ethmoid, CRSsNP, and CRSwNP ethmoid. Two-sided Wilcoxon tests were used for significance. Boxes indicate interquartile range (25th–75th), with the lines indicating the median. (D) Scatterplots of *CCL13* mRNA levels vs. eosinophil spatial signatures in the GeoMx data. Points are colored by CD45^+^ (magenta) and PanCK^+^ (yellow) regions. Blue lines showed regression fit; gray bands indicated 95% confidence interval (CI). Regression indices and *p* values shown on plot. (E) Scatterplots illustrating the correlation between *CCL13* expression levels and eosinophil signature scores in CD45^+^ or PanCK^+^ regions of GeoMx spatial transcriptomics acquisition, with sample origins color-coded to represent CRS NPs (purple) and healthy control samples (orange). Diagrams above the scatterplots indicate regions where *CCL13* and eosinophil spatial gene signatures were captured. (F) Representative multiplexed immunofluorescence images from GeoMx spatial profiling of CRSwNP (top) and control (bottom) tissues, with zoomed insets showing IMM cell infiltration (red arrows) into the EPI region. Scale bars: 200 μm (left) and 50 μm (right). (G) The proposed model in which macrophages secreting *CCL13*/*CCL18* chemokines attract eosinophils to infiltrate the epithelium in CRS NPs. (H) Representative H&E staining images of a CRSsNP (upper) and a CRSwNP sample (lower). Red arrows highlight eosinophil infiltration into the EPI region. (I) UMAP plot depicting subtypes and corresponding annotations of MCs in CRS and control samples. (J) L-R interactions identified between MCs and CD4^+^ T cells in CRSwNP (purple) and CRSsNP (green). L-R pairs with purple bars crossing the 0.5 dotted line indicate predominance in CRSwNP, while those with green bars crossing the dotted line indicate predominance in CRSsNP. Significant interactions are color-coded accordingly (*p* < 0.05, Wilcoxon test, two-sided). L-R labels from CellChatDB. (K) Dot plot demonstrating the significance and strength of *IL4*/*IL13*-related L-R interactions between MCs and CD4^+^ T cells in CRSwNP (purple) and CRSsNP (green), with circle size indicating significance and color intensity indicating interaction probability.

Given the postulated role of myeloid cells (MLCs) in CRS,^10,14,17^ we further stratified the MLC cluster into subtypes, including macrophages, monocytes, and DCs (Figure 2A). These subtypes were represented across the non-CRS and CRS samples (Figure S2A). We quantified the percentage composition of the three main subtypes of macrophages identified (*CCL4L2*, *MRC1*, or *VEGFA* high) (Figure S1D). While proinflammatory (*CCL4L2* high) macrophages did not vary in numbers significantly, immunoregulatory macrophages (*MRC1* and *VEGFA* high) were significantly elevated in CRSwNP, compared with non-CRS controls or CRSsNP (Figure S1D). A similar analysis was performed for the other MLCs without any notable differences (Figure S1D). These results are consistent with a predicted dysregulation in macrophage cell states in CRSwNP. We next performed differential gene analysis to identify differentially expressed genes (DEGs) in the macrophages in CRSsNP and CRSwNP tissue (Figure 2B). Among them were genes associated with antigen presentation, complement pathway activation, and chemokines linked to IMM cell recruitment and activation. Scoring of M1- and M2-like activity through a previously curated set of genes^18,19^ confirmed the increased frequency of immunoregulatory macrophages in polyp tissues from the scRNA-seq data (Figure 2C) and the opposite for proinflammatory macrophages (Figure S2B). Additionally, we repeated the analysis on scRNA-seq CRS data of control, CRSsNP, and eosinophilic CRSwNP samples, as described previously,^17^ and observed similar conclusions (Figure S2C), orthogonally validating our observations. We additionally scored for M2-like macrophagerelated genes in our spatial transcriptomics data and confirmed the significant elevation of M2-like gene expressions in CD45-positive regions of CRSwNP samples compared with CRSsNP and controls (Figure S2D). These results confirm an increase in immunoregulatory macrophages in CRSwNP.

### Macrophage recruitment of eosinophils in CRS occurs through *CCL13* and *CCL18*

Given the established role of C-C Motif Chemokine Ligand 13 (CCL13) and CCL18 for the recruitment of monocytes, including eosinophils,^20,21^ and their significant transcriptional upregulation within macrophages in our CRSwNP samples and previous studies^14,17^ (Figures 2B, S2E, and S2F), we first confirmed that eosinophils were increased in NP tissue, as compared with control ethmoid tissues, via spatial transcriptomics (Figure S2G). This allowed the *in situ* assessment of the intact CRS tissue microenvironment, given that single-cell dissociation approaches can often result in the loss of delicate cell types.^22^ We next tested the hypothesis that *CCL13* and *CCL18* were involved in the recruitment of eosinophils.^14^ From the spatial transcriptomics data, we observed significant correlations in the expression of both tissue location-based pairwise spatial analysis of these signatures, indicating potential dynamics of eosinophil recruitment by macrophages (Figure 2D). We observed a statistically significant correlation between *CCL13* and *CCL18* expression with the influx of eosinophils in the pan-cytokeratin (PanCK)-positive EPI but not CD45-positive IMM regions (Figures 2E and S2I). Representative immunofluorescence images from the tissues stained for GeoMx Digital Spatial Profiler (GeoMx), and regions defined for spatial transcriptomics data collection, further confirmed the localization of IMM cell infiltration into the EPI regions in CRSwNP but not the healthy controls (Figure 2F). Taken together, these correlative observations suggest a potential model in which CCL13/CCL18-secreting macrophages in CRSwNP may be involved in eosinophil recruitment into the nasal epithelium (Figure 2G).^14^ We additionally confirmed these observations from the H&E staining of these tissues for histological evidence of eosinophilic infiltration (Figure 2H). We then performed pathology eosinophil infiltration scoring on H&E-stained slides by quantifying the number of eosinophils located in or near EPI regions per high-power field (HPF, 200× magnification). Nearly half (48%) of CRSwNP samples exhibited heavy eosinophil infiltration in EPI regions, significantly higher than that observed in CRSsNP and control samples from 113 individuals (Figure S2J). Together, these indicate eosinophil recruitment by macrophages through the CCL13 and CCL18 axis in CRSwNP.

### MC enrichment promotes type 2 IMM responses in NPs

Given the intricate relationship between MCs and the type 2 IMM response in T cells, we next sought to better understand their roles in CRSwNP disease.^9^ We observed two major subtypes of MCs, as stratified by MCs expressing tryptase (TPSAB1) without chymase (CMA1): MCT, with high expression of IL-17 receptor B (IL-17RB; MCT_IL-17RB), and MCs with high expression of the protease tryptase (MCTC), along with cathepsin G and chymase (CTSG; MCTC_CTSG) (Figure 2I). We postulated these subtypes to have distinct roles in the recruitment and interaction with key immunological players within the CRSwNP tissue microenvironment. We explored this hypothesis via ligand-receptor (L-R) analysis to identify several pathways related to IMM and tissue remodeling and CD4^+^ T cells, such as *IL2*, *OX40*, *CCL*, *EPHB*, *PROS*, *IL4*/*IL13*, *PARs*, *CD22*, *ICAM*, *SEMA7*, *LIFR*, *CLEC*, and *OSM* (Figure 2J). Of particular interest were the key mediators in type 2 inflammation: *IL4* and *IL13* (Figure 2K), which are expressed predominantly by MCs in our study (Figure S2K) and are implicated in MC and CD4^+^ T cell interactions in CRSwNP but not in CRSsNP, with the *CSF2* signaling pathway as a control.

### Identification of key players in the IMM-EPI crosstalk and remodeling in CRSwNP

**Figure 3.**
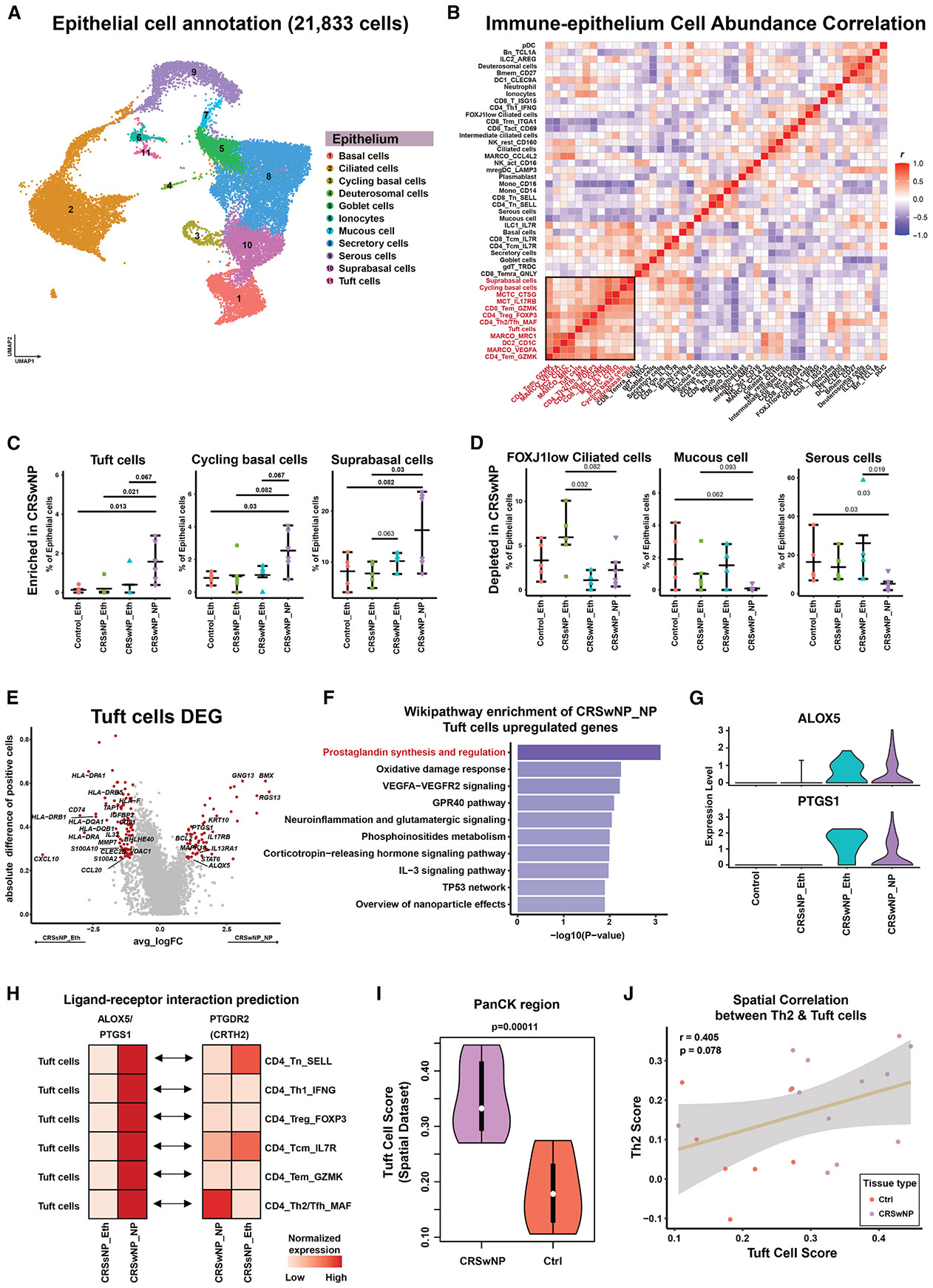
Tuft cells and other EPI populations mediate IMM cell recruitment in CRSwNP (A) UMAP of 21,833 EPI cells including basal, ciliated, cycling basal, goblet, tuft, and other specialized types. (B) Correlation heatmap of IMM and EPI cell abundances. The black box highlights a key cluster of correlated populations. (C) Quantification of cell populations significantly enriched in CRSwNP NPs, showing increased frequencies of tuft cells, cycling basal cells, and suprabasal cells, compared with control and CRSsNP samples. Statistical significance was determined using two-sided Wilcoxon tests. Bars represent the data range, with the lines indicating the median. (D) Quantification of cell populations depleted in CRSwNP NPs, demonstrating reduced frequencies of FOXJ1low ciliated cells, mucous cells, and serous cells. (E) Volcano plot showing DEGs in tuft cells between CRSwNP and CRSsNP conditions. Red dots indicate significantly altered genes with ∣logFC∣ > 1 and expressing percentage (pct) > 0.25. (F) Pathway enrichment analysis of genes upregulated in CRSwNP tuft cells, highlighting prostaglandin synthesis and regulation as the top enriched pathway. (G) Violin plots showing expression levels of prostaglandin pathway genes ALOX5 and PTGS1 across control and CRS samples. (H) Heatmap depicting predicted L-R interactions between tuft cells and CD4^+^ T cell subsets, focusing on ALOX5/PTGS1 and PTGDR2 signaling. (I) Violin plots comparing tuft cell spatial signature scores between CRSwNP and control samples in PanCK^+^ EPI regions from spatial transcriptomics data. Boxes indicate interquartile range (25th–75th), with the dot indicating the median. (J) Scatterplot demonstrating the spatial correlation between tuft cell and Th2 cell signature scores, suggesting functional interaction in CRSwNP. The brown line shows the regression fit; the gray band indicates the 95% confidence interval (CI). Regression indices and *p* values shown on the plot. See also Figure S3.

Aside from the IMM populations resolved in our single-cell transcriptomics, we also delineated EPI cell types. We identified 11 EPI cell types (21,833 cells total) across non-CRS and CRS samples, including secretory, ciliated, basal, goblet, tuft, and others (Figure 3A). EPI origins were visualized in a UMAP plot by tissue type and individuals (Figure S3A), with canonical markers shown across the repertoire (Figure S3B). Differential gene expression across IMM and EPI cell types further revealed the complex cellular composition and states in CRS tissues (Figure 1D).

Building on our data and prior evidence of IMM-EPI remodeling in diseases,^5,6^ including CRSwNP,^4^ we quantified cell abundance correlations across IMM and EPI subsets. A key cluster of EPI and IMM cell types showed strong correlation, suggesting potential crosstalk in CRSwNP (Figure 3B; black box). Tuft cells, cycling basal cells, and suprabasal cells were enriched in CRSwNP polyps, while FoxJ1-low ciliated, mucous, and serous cells were depleted (Figures 3C, 3D, and S3C). These trends suggest that tuft and basal cells may drive IMM-EPI interactions and attract IMM infiltrates in chronic inflammation and NP formation.

To test this, we performed DEG analysis of tuft cells across disease types, identifying multiple signaling pathways (e.g., G protein, tyrosine kinase, mitogen-activated protein kinase [MAPK]), anti-apoptotic genes (*BCL2*), and cytokine pathways (*IL17RB*, *IL13RA1*, *STAT6*) upregulated in CRSwNP (Figure 3E). Conversely, antigen-presentation components were upregulated in CRSsNP, pointing to distinct tuft cell states. Additional pathways enriched in CRSwNP tuft cells included the prostaglandin pathway (Figure S3D), an inflammatory pathway not previously described in CRS. Gene set enrichment analysis confirmed prostaglandin pathway activation in CRSwNP (Figure 3F), with expression of *ALOX5* and *PTGS1* in CRSwNP polyps and ethmoid tissue (Figure 3G). Reanalysis of prior studies confirmed similar tuft cell trends (Figures S3E and S3F).

L-R analysis revealed tuft cell interactions with Th2 CD4^+^ T cells and depletion of naive and central memory CD4^+^ T cells in CRSwNP (Figure 3H), consistent with abundance correlations (Figure 3B). Spatial transcriptomics confirmed increased tuft cell density in the CRSwNP epithelium *in situ* (Figure 3I) and a positive spatial correlation (r = 0.405, *p* = 0.078) between tuft cells in the PanCK^+^ region and Th2 CD4^+^ T cells in CD45^+^ regions of CRSwNP tissue (Figure 3J). Together, these findings implicate chemosensory tuft cells as mediators of IMM cell recruitment, including Th2 CD4^+^ T cells, into the CRSwNP microenvironment, potentially contributing to type 2 inflammation.

### A basal cell trajectory drives EPI-IMM remodeling in NP formation

**Figure 4.**
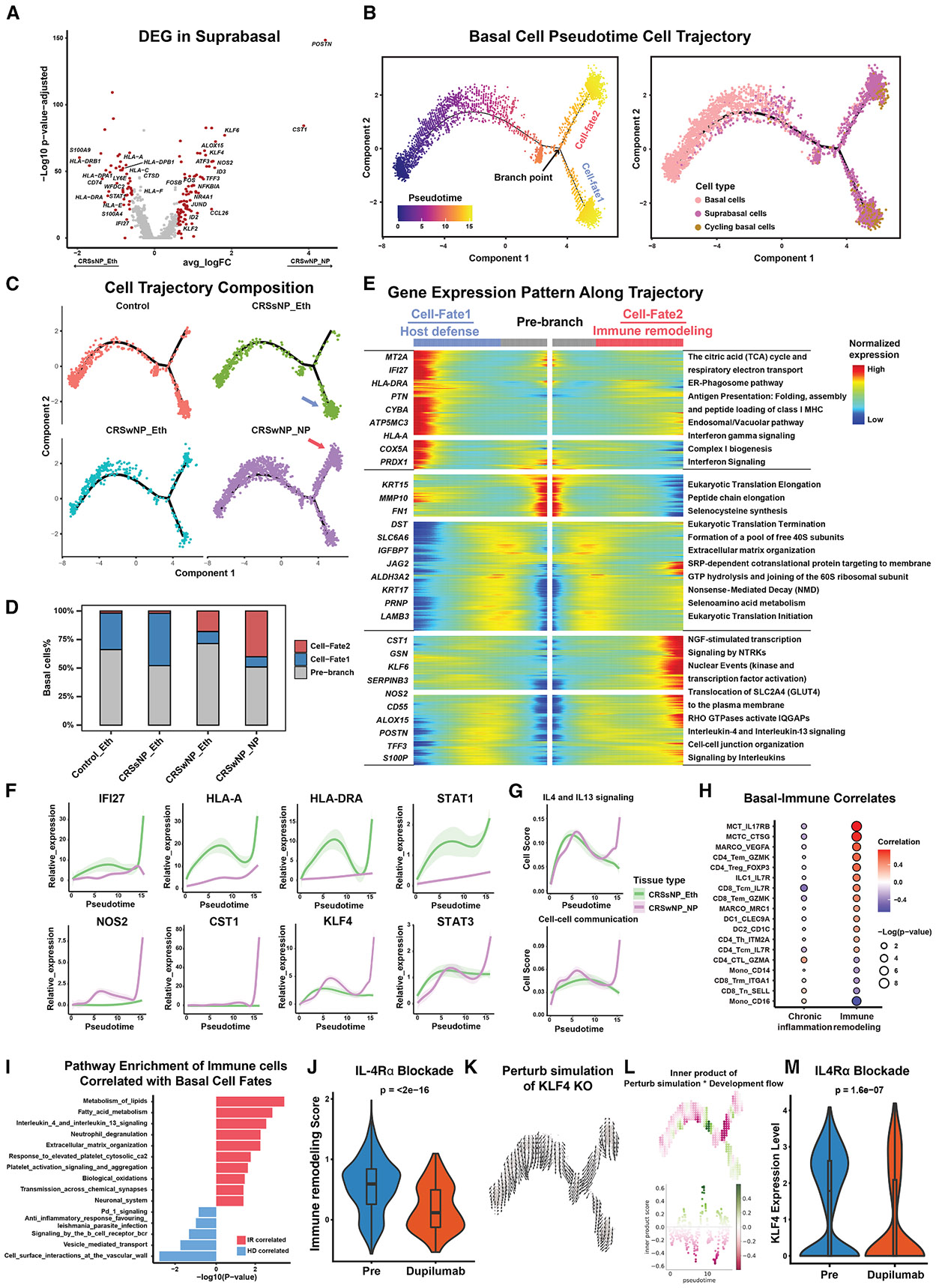
Basal cell developmental trajectories drive tissue remodeling in CRSwNP (A) Volcano plot showing DEGs in suprabasal cells, comparing CRSsNP and CRSwNP conditions. Red dots indicate significantly altered genes (∣log_2_FC∣ > 1.5). (B) Pseudotime trajectory analysis of basal cell differentiation using Monocle2, colored by pseudotime progression (left) and cell-type identity (right), revealing a bifurcation point leading to two distinct cell fates. (C) Cell trajectory plots showing the distribution of basal cells from control and CRS samples along the pseudotime trajectory. (D) Quantification of cell fate distributions across sample types, demonstrating enrichment of Cell-fate2 (IMM remodeling) in CRSwNP NPs. (E) Heatmap showing gene expression dynamics along the basal cell trajectory, with distinct gene modules characterizing pre-branch, Cell-fate1 (host defense), and Cell-fate2 (IMM remodeling) states. Associated enriched pathways and their annotations are shown on the right. (F) Expression dynamics of key genes during pseudotime progression in CRSsNP (green) and CRSwNP (purple) conditions, highlighting differential regulation of inflammatory and developmental pathways. (G) Dynamic expression scores for *IL4/IL13* signaling and cell-cell communication pathways along the basal cell pseudotime trajectory. (H) Correlation analysis between basal cell states and IMM cell populations, showing distinct IMM cell associations with host defense, compared with IMM remodeling cell fates. (I) Pathway enrichment analysis of IMM cells correlated with different basal cell fates. (J) Violin plots showing a reduction in the IMM remodeling signature following dupilumab treatment. Boxes indicate interquartile range (25th–75th), with the lines indicating the median. (K) *In silico* perturbation analysis of KLF4 knockout showing altered developmental trajectories as indicated by the arrows. (L) Quantification of the simulated perturbation effects in (K). (M) A reduction of KLF4 expression is observed following dupilumab treatment. Boxes indicate interquartile range (25th–75th), with the lines indicating the median. See also Figures S4 and S5.

We next investigated the role of basal cells, a critical node in CRSwNP EPI-IMM remodeling identified in our analysis (Figures 3B and 3C). We observed differential expression between suprabasal and cycling basal cells (Figures 4A and S4A), with a sizable overlap of genes upregulated in CRSwNP vs. CRSsNP across both basal cell states (Figure S4B). Given the proliferative and developmental potential of basal cells, we postulated that trajectory analysis could better capture their differentiation states. Pseudotime analysis confirmed that undifferentiated basal cells appear earlier, followed by a bifurcation point in the trajectory, initially termed Cell-fate1 and Cell-fate2 (Figure 4B). Basal cells from individuals with CRSwNP were enriched in Cell-fate2, while those from control and CRSsNP tissues were more represented in Cell-fate1 (Figures 4C and 4D), suggesting divergent outcomes for basal cell differentiation in CRSwNP vs. CRSsNP upper airway environments.

DEG analysis stratifying Cell-fate1 and Cell-fate2 revealed differences in gene programs (Figures 4E and 4F), including IL-4/IL-13 signaling and cell-cell communication in CRSwNP (Figures 4E and 4G), vs. elevated interferon (IFN) signaling and antigen presentation in CRSsNP (Figures 4E and S4C). Cell-fate2 also showed upregulation of metabolic, IMM attractant, and remodeling pathways (Figure 4E), with this signature enriched in CRSwNP spatial data (Figure S4D). We further found Cell-fate2 basal cells correlated with eosinophils in spatial transcriptomics (Figure S4E). Given their linked roles, we renamed Cell-fate1 to “host defense” and Cell-fate2 to “IMM remodeling” (Figure 4E).

We cross-validated host defense and IMM remodeling transcriptional signatures using public CRS scRNA-seq data,^17^ confirming consistent enrichment of host defense in CRSsNP and IMM remodeling in CRSwNP (Figure S4F). We also observed increased basal IMM interactions in IMM remodeling-directed basal cells (Figure 4H) and upregulation of metabolism, IL-4/IL-13 signaling, neutrophil degranulation, and tissue remodeling pathways (Figure 4I). We next assessed basal cell development in relation to IMM cell dynamics via a basal transitional continuum analysis.^23^ Principal-component analysis (PCA) of DEGs in basal cells revealed a trajectory across control, CRSsNP, and CRSwNP samples (Figure S5A). Plotting IMM cell fractions along this continuum showed trends in IMM representation: memory CD4^+^ and CD8^+^ T cells (CD4_Tcm_IL-7R, CD8_Tcm_IL-7R), innate lymphoid cells (ILC1_IL-7R), macrophages (MARCO_MRC1), MCs (MCT_IL-17RB), and specific T cell subsets (CD4_Th_ITGM2A, CD4_CTL_GZMA, CD8_Trm_ ITGA1) showed distinct enrichment patterns (Figure S5B). As noted previously, several IMM populations were more represented in CRSwNP (Figures 1 and S1), particularly at later stages of the basal continuum. These findings reinforce the IMM remodeling trajectory of CRSwNP basal cells and suggest that their differentiation may influence IMM recruitment and regulation. Altogether, this points to basal cells being pushed toward a state primed for IMM attraction and remodeling—implicating their trajectory as a potential determinant of NP formation. Future experimental systems will be needed to functionally validate this basal differentiation pathway in CRS pathogenesis.

### A reduction in the Cell-Fate2 basal cell trajectory upon use of immunotherapeutics intervention for CRSwNP

The upregulation of *IL4* and *IL13* in CRSwNP and in the basal IMM remodeling trajectory supports the pivotal role of these basal cells in CRSwNP and NP development. We confirmed that IL-4 and IL-13 cytokine stimulation of non-NP-derived basal cells^10^ increased the IMM remodeling cell fate transcriptomic signature (Figure S4G). Dupilumab is an IL-4/IL-13 receptor alpha antagonist that is approved by US food and drug administration (FDA) as an add-on maintenance treatment in adult individuals with inadequately controlled CRSwNP.^24,25^ Inferior turbinate and NP tissues sampled pre- and post-dupilumab treatment for scRNA-seq were reanalyzed,^10^ and they showed a statistically significant reduction in the IMM remodeling transcriptomic signatures when comparing basal cells pre- and post-dupilumab treatment (Figure 4J). Given the differential enrichment of the transcription factor (TF) Kruppel-Like Factor 4 (KLF4) in the IMM remodeling cell fate (Figure 4F), we performed an *in silico* perturbation analysis to examine its role in basal cell development with CellOracle,^26^ which computationally predicts cellular state trajectories when specific TF is knocked out using gene regulatory networks. The *in silico* KLF4 knockout confirmed altered developmental trajectories specific for the IMM remodeling basal cell fate (Figure 4K), with the inner product analysis showing negative perturbation scores, supporting a hypothesis in which KLF4 loss inhibits or reverses differentiation of basal cells toward the IMM remodeling fate (Figure 4L). Supporting the functional significance of this TF, we observed reduced KLF4 expression in basal cells following dupilumab treatment (Figure 4M). These results potentially implicate basal cells and the IMM remodeling developmental trajectory as important components for EPI-IMM interactions and remodeling in NP formation in individuals with CRS.

### Conserved IMM-EPI remodeling tissue features in a large-scale spatial transcriptomics validation cohort

**Figure 5.**
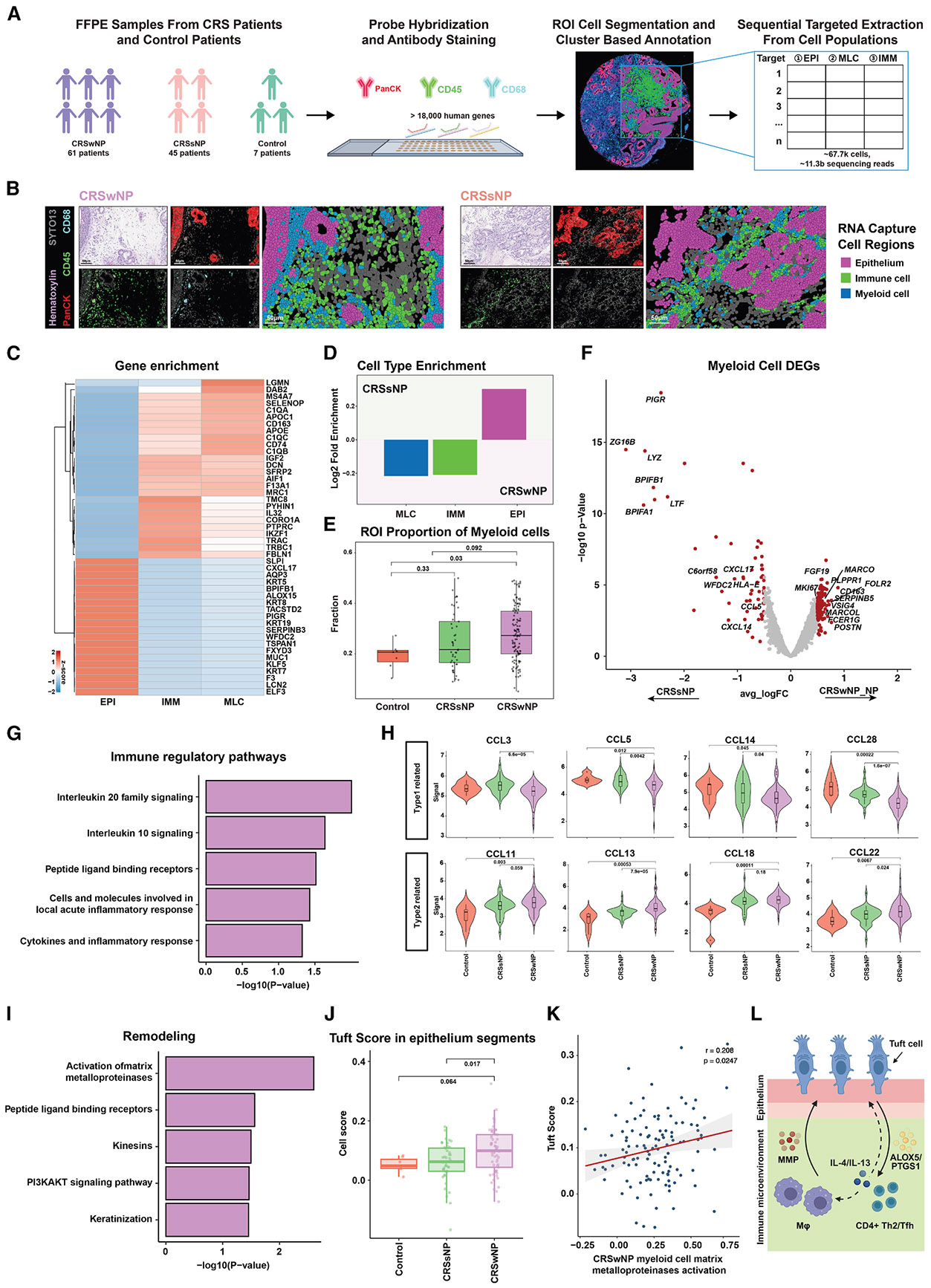
Large-scale spatial transcriptomics reveal conserved IMM-EPI remodeling features in CRSwNP (A) Experimental workflow for spatial transcriptomics analysis of a large validation cohort comprising 61 CRSwNP, 45 CRSsNP, and 7 control individuals, using GeoMx spatial transcriptomics coupled with PanCK, CD45, and CD68 antibodies to guide whole-transcriptome capture of specific cell types. (B) Representative images showing H&E staining, multiplexed immunofluorescence, and annotated cell regions for CRSwNP (left) and CRSsNP (right) samples. Cell types are annotated based on the multiplexed antibody stainings as EPI cells (magenta), IMM cells (green), and MLCs (blue). Scale bars: 50 m. (C) Heatmap demonstrating cell-type-specific gene expression patterns across EPI, IMM, and MLC regions. (D) Quantification of cell-type enrichment showing differential distribution of MLCs and IMM and EPI cells between CRSsNP and CRSwNP samples. (E) Barplot comparing the proportion of MLCs across control, CRSsNP, and CRSwNP samples. A pairwise two-sided Wilcoxon test was performed. See also Figure S6. Boxes indicate interquartile range (25th–75th), with the lines indicating the median. (F) Volcano plot showing DEGs in MLC regions between CRSsNP and CRSwNP conditions. Significant genes are highlighted in red (∣log_2_FC∣ > 1.5). (G) REACTOME pathway analysis highlight IMM regulatory pathways enriched in CRSwNP MLCs. (H) Violin plots comparing expression of type 1 (top row) and type 2 (bottom row) chemokines across sample types, demonstrating distinct inflammatory signatures. Boxes indicate interquartile range (25th–75th), with the lines indicating the median. (I) REACTOME pathway analysis showing tissue remodeling pathways enriched in CRSwNP MLCs. (J) Barplot comparing tuft cell signature scores across control, CRSsNP, and CRSwNP EPI regions. Boxes indicate interquartile range (25th–75th), with the lines indicating the median. (K) Correlation analysis between tuft cell scores and matrix metalloproteinase activation in MLCs. Blue lines indicate the mean, and gray regions highlight the 95% CIs. (L) Proposed model illustrating the interaction between tuft cells, MLCs, and CD4^+^ T cells in tissue remodeling and type 2 inflammation. See also Figure S2.

To determine the generalizability and clinical relevance of our findings, we assembled a large cohort of nasal tissues from 61 CRSwNP, 45 CRSsNP, and 7 control individuals (Figure 5A). We applied antibodies against PanCK, CD45, and CD68, along with the Nanostring GeoMx Whole Transcriptome Atlas probeset (>18,000 genes). We performed region of interest (ROI) selection for each individual’s tissue (Figure 5A), followed by single-cell segmentation and annotation of the images (stratified as EPI cell, IMM cell, or MLC types), for downstream transcriptome extraction from each ROI (Figure 5B) as previously described.^27^ Batch effects were corrected as described in STAR Methods, with results confirming the stratification of non-CRS, CRSsNP, and CRSwNP samples, along with the three cell-type-specific transcriptomes (Figures S6A and S6B). We first validated capture specificity of EPI, IMM, and MLC transcriptomes via DEG analysis, confirming distinct expression across EPI and IMM cells and MLCs, using canonical markers such as keratins and IMM regulators and genes like *CD163* and *C1QA*, along with additional confirmation of Th1/Th2, immunoregulatory macrophage, and eosinophil enrichments (Figures 5C and S6C-S6E). We then performed single-cell (Figure 5A) spatial analysis on the multiplexed antibody images. Cell-type enrichment analysis showed CRSsNP tissues had more EPI cells, while CRSwNP had greater IMM and MLC enrichment (Figure 5D). We also confirmed increased MLC proportions from control to CRSsNP to CRSwNP, consistent with our scRNA-seq findings (Figures 5E, S1D, and S5B). Spatial DEG analysis of the MLC compartment showed upregulation of IMM regulatory and remodeling genes in CRSwNP, including M2-like immunosuppressive genes (*MACRO*, *CD163*, *MACROL*), while *LYZ* and *BPIFB1* were enriched in CRSsNP (Figure 5F).

Building on the MLC analyses, pathway enrichment revealed upregulation of IMM regulatory pathways in CRSwNP MLCs, including IL signaling and peptide ligand binding receptors (Figure 5G). Chemokine profiling revealed differential expression between type 1 (*CCL3*, *CCL8*) and type 2 (*CCL13*, *CCL18*, *CCL22*) responses, consistent with a type 2 bias in CRSwNP and type 1 in CRSsNP (Figure 5H).

Consistent with observed remodeling in CRSwNP, MLCs emerged as key participants in EPI-IMM crosstalk, marked by upregulated pathways for matrix metallopeptidase (MMP) activation, peptide ligand receptors, kinesins, phosphatidylinositol 3-kinase-Ak strain transforming (PI3K-AKT) signaling, and keratinization (Figure 5I). We confirmed increased tuft cell enrichment in CRSwNP seen in scRNA-seq (Figure 3C) in the expanded spatial cohort (Figure 5J). Notably, tuft cell enrichment correlated positively with MLC MMP activity (Figure 5K), suggesting potential functional interactions during remodeling and a possible vulnerability in CRSwNP. These data support a model in which tuft cells, CD4^+^ Th2 cells, and MLCs cooperatively remodel the CRSwNP tissue microenvironment (Figure 5L).

### Large-scale validation of IMM-EPI remodeling in CRSwNP

**Figure 6.**
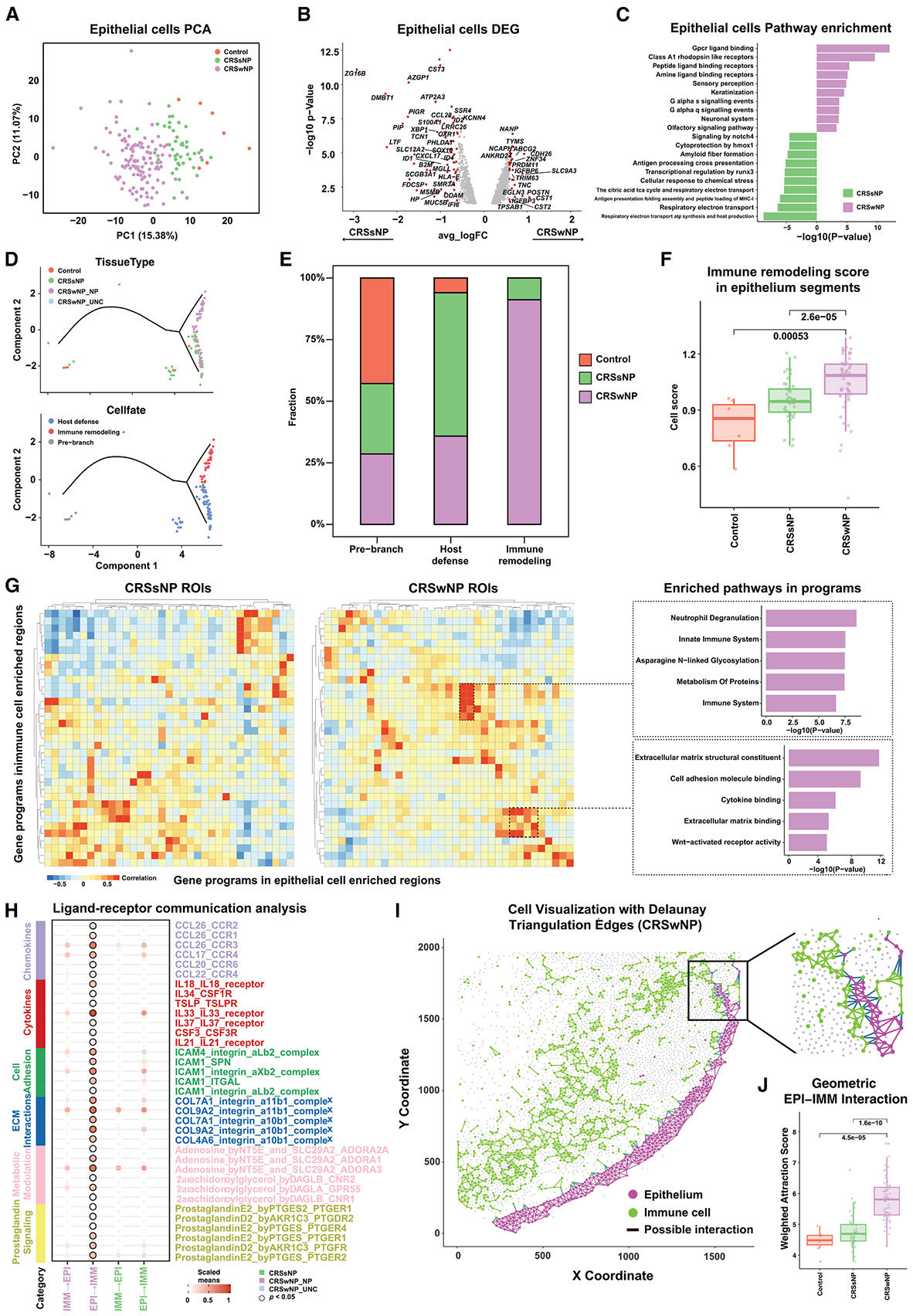
Spatial transcriptomics identifies of IMM-EPI remodeling and associated pathways in CRSwNP (A) PCA of EPI regions using top 500 variable genes, demonstrating distinct clustering of control, CRSsNP, and CRSwNP samples. The first two principal components and their eigenvalues are shown, accounting for 15.38% and 11.77% of the variance. Samples are colored based on the origin of tissue types. (B) Differential gene expression analysis of EPI regions comparing CRSsNP and CRSwNP, with significant (∣fold change log_2_FC∣ > 1.5) genes highlighted in the volcano plot in red. (C) REACTOME pathway analysis revealing enrichment of GPCR signaling, L-R interactions, and keratinization pathways in CRSwNP epithelium, while antigen presentation and metabolic pathways are enriched in CRSsNP. (D) Projection of EPI ROIs onto the basal cell trajectory space, colored by tissue type (top) and cell fate (bottom), demonstrating conservation of the developmental trajectories identified in single-cell analysis. (E) Quantification of cell fate distributions across samples, showing enrichment of IMM remodeling fate in CRSwNP. (F) Barplot comparing IMM remodeling signature scores across control, CRSsNP, and CRSwNP EPI regions, confirming increased remodeling in CRSwNP, using a two-sided Wilcoxon test. Boxes indicate interquartile range (25th–75th), with the lines indicating the median. (G) Heatmaps showing co-expressed gene programs between IMM and EPI regions in CRSsNP (left) and CRSwNP (right) samples, with functional pathway enrichment analysis below highlighting differences in IMM and tissue remodeling processes. (H) Dot plot analysis of L-R pairs mediating EPI-IMM communication, showing enrichment of type 2 inflammatory signaling specifically in CRSwNP. Interactions are grouped by functional categories (chemokines, cytokines, ECM-receptor interactions, and prostaglandin signaling). (I) Spatial visualization using Delaunay triangulation showing organization of EPI (magenta) and IMM (green) cells in CRSwNP tissue, with cellular interactions indicated by edges. Each dot represents an individual cell, and the edges between them indicate a proximity of 50 μm. Edges connecting cells within the same type are colored to match the nodes, while edges spanning across different cell types are shown in blue. (J) Quantification of geometric interaction scores between EPI and IMM cells, demonstrating increased spatial association in CRSwNP, compared with control and CRSsNP samples. Statistical significance was assessed using a two-sided Wilcoxon rank-sum test, with *p* values adjusted via Bonferroni correction. Boxes indicate interquartile range (25th–75th), with the lines indicating the median. See also Figure S6.

PCA of EPI regions, using the top 500 variable genes, revealed systematic differences in composition and gene programs across non-CRS, CRSsNP, and CRSwNP individuals (Figures 5A, 6A, S6A, and S6B). DEG analysis comparing CRSsNP and CRSwNP EPI cells identified multiple DEGs (Figure 6B), with pathway analysis showing enrichment of G protein-coupled receptor (GPCR) signaling, L-R interactions, and keratinization in CRSwNP, while antigen presentation and metabolic pathways were upregulated in CRSsNP (Figure 6C), supporting prior findings (Figure 4E).

We projected EPI transcriptomes from each individual’s tissue ROI onto the basal cell trajectory from scRNA-seq (Figures 4B and 4C), revealing a similar distribution: CRSsNP samples enriched in the host defense fate and CRSwNP in IMM remodeling (Figures 6D and 6E), consistent with our single-cell data (Figures 4B-4D). Quantification confirmed this trend (Figure 6E), with significantly increased IMM remodeling signatures in CRSwNP EPI cells relative to non-CRS and CRSsNP samples (Figure 6F). These results support our observation that eosinophil infiltration correlates with *CCL13* and *CCL18* expression (Figures 2D-2G and S6F).

IMM cell analyses also revealed distinct transcriptional programs (Figure S6G) and pathway enrichments (Figure S6H), differentiating CRSwNP from CRSsNP. We identified co-expressed IMM-EPI gene modules stratifying subtypes (Figure 6G), with enrichment pointing to neutrophil degranulation, glycosylation, and extracellular matrix (ECM) adhesion pathways.

Finally, L-R analysis uncovered consistent type 2 inflammatory signaling specific to EPI-IMM crosstalk in CRSwNP but not in CRSsNP (Figure 6H). Spatial mapping via Delaunay triangulation highlighted complex cellular organization in CRSwNP tissue (Figure 6I), and geometric analysis confirmed elevated EPI-IMM interactions in CRSwNP relative to other groups (Figure 6J). Together, these findings support a model of IMM-EPI remodeling involving basal cells, type 2 T cells, and MLC populations in CRSwNP.

## DISCUSSION

Our integrated single-cell and spatial transcriptomic analysis reveals coordinated IMM-EPI interactions that shape CRSwNP pathogenesis. Across tissue compartments, CD4^+^ T cells exhibit heightened type 2 inflammatory activation (Figure 1); macrophages polarize to an immunoregulatory state in CRSwNP, secreting eosinophil-attracting chemokines CCL13 and CCL18 (Figures 2A-2D), which spatially correlates with eosinophil signatures in EPI regions (Figures 2E-2I). MCs are enriched in CRSwNP (Figures 2I-2K), with IL-4/IL-13-mediated interactions with T cells likely reinforcing type 2 inflammation. We identified two distinct MC subsets (EPI MCT_IL-17RB and subepithelial MCTC_CTSG) with divergent immunomodulatory profiles. Tuft cells emerge as EPI regulators that express prostaglandin synthesis enzymes ALOX5 and PTGS1 (Figures 3E-3G), potentially recruiting Th2 cells via prostaglandin D2 receptor 2 (PTGDR2), and co-localize spatially with Th2 cells in polyps (Figures 3I and 3J). Finally, basal progenitors in CRSwNP exhibit an aberrant differentiation trajectory skewed toward an IMM remodeling fate (vs. a “chronic inflammation” host defense fate) (Figures 4B-4E), a transition enhanced by IL-4/IL-13 signaling and attenuated by IL-4Rα blockade (dupilumab) (Figures 4K-4M), with KLF4 as a key transcriptional regulator.

These findings were further validated in a large-scale spatial transcriptomics cohort of over 100 individuals (Figures 5 and 6), which recapitulated IMM-EPI remodeling features of CRSwNP. Spatial profiling confirmed distinct EPI-IMM gene modules, L-R interactions, and tissue remodeling programs that were largely unique to CRSwNP and absent in CRSsNP (Figures 6G-6K). Importantly, this cohort included samples from North America and Asia, reflecting transcriptomic heterogeneity. PCA revealed only partial separation between CRSwNP and CRSsNP (Figure 6A), and gene modules in CRSwNP included both eosinophilic and neutrophilic inflammatory signatures (e.g., neutrophil degranulation) (Figure 6H). While our current analysis focused on consistently observed EPI-IMM interactions in CRSwNP, future studies will explore regional and endotypic heterogeneity.

This work illustrates the power of integrating scRNA-seq and GeoMx Digital Spatial Profiler (DSP) spatial transcriptomics to dissect tissue-level inflammation in CRS. Our high-dimensional dataset complements prior efforts using single-cell^10,17,28^ and spatial^29^ approaches and forms a valuable basis for hypothesis generation. Functional validation via *in vitro* or *in vivo* perturbation models will be essential to confirm causality. Despite limitations related to sample composition and cell recovery, our analytic framework balances statistical power with cohort interpretability and supports translational directions. From a therapeutic standpoint, our findings suggest promising targets, including the following: (1) immunoregulatory macrophages or their chemokines (CCL13/CCL18), (2) IL-4/IL-13-driven MC-T cell signaling, (3) prostaglandin-mediated tuft-Th2 cell crosstalk, and (4) KLF4-regulated basal cell fate decisions. Dupilumab’s efficacy in reducing the IMM remodeling basal state provides a strong proof of concept of such a discovery platform.

In conclusion, we present an extensive IMM-EPI data resource of the CRS tissue environment, offering a framework for mechanistic discovery and therapeutic innovation in upper airway inflammation.

### Limitations of the study

This study was conducted using observational, retrospective clinical tissue samples, which hinders our ability to infer causality. Our identification of key IMM-EPI interactions and basal cell trajectories associated with disease states is largely observational because the lack of suitable animal or *in vivo* models limits the experimental validation possible, including gain- or loss-of-function studies; our findings are thus largely correlative. Our integrative multi-omics discovery framework revealed novel insights into CRSwNP pathophysiology, but the translational potential remains to be validated in clinical contexts. Finally, limitations inherent to single-cell dissociation and spatial profiling, including incomplete cell-type recovery and regional tissue heterogeneity, may introduce bias. Nonetheless, we applied rigorous statistical and computational approaches to ensure the robustness of our inferences, with our findings validated across a multi-institutional cohort. Future functional studies will be essential to confirm mechanistic roles of identified pathways and cell states and their translational potential.

## STAR★METHODS

### EXPERIMENTAL MODEL AND STUDY PARTICIPANT DETAILS

Individuals were diagnosed with CRSwNP and CRSsNP based on the European position paper on rhinosinusitis and nasal polyps (EPOS) 2012 and International Consensus of Allergy and Rhinology: Rhinosinusitis (ICAR:RS) guidelines.^42^ CRSwNP, CRSsNP, and controls were recruited from Stanford University, Dokkyo University and National Taiwan University. Tissues from the ethmoid sinus mucosa or nasal polyps were collected during endoscopic sinus surgery. Five control individuals underwent skull base surgery requiring ethmoid sinus surgery for treatment of cerebrospinal fluid leak, meningioma, or pituitary adenoma. None of the control individuals had evidence of CRS or other upper airway inflammatory diseases on CT/MRI radiography or endoscopy. Individuals with unilateral sinus disease, fungal or allergic fungal rhinosinusitis, antrochoanal polyps, cystic fibrosis, aspirin-exacerbated respiratory disease, or paranasal sinus cysts were excluded from this study. Individual characteristics, including demographics, medical history, and past medication use were collected. Individual data, including medication history, were independently verified through direct interview by a trained research technician/physician and by a questionnaire additionally administered on the day of surgery to confirm accuracy of existing records derived from the individual’s electronic medical or pharmacy. In particular, to avoid confounders in the epithelial/immune cell findings associated with use of common anti-inflammatory medications in CRS, all included CRSsNP and CRSwNP individuals were devoid of oral prednisone/methyl-prednisolone exposure and higher dose topical budesonide and mometasone nasal irrigations x 4 weeks, as well as lower-dose topical nasal steroid sprays such as fluticasone and mometasone for 2 weeks, prior to ethmoid or NP tissue sampling. Antibiotic use within 4 weeks of surgery also led to exclusion. Any doubt in individuals medication use led to exclusion from final analysis. Individual characteristics are shown in Table S1. The study complied with the Declaration of Helsinki and all relevant ethical regulations of each institution, and written informed consent was obtained from each individual under approved Institutional Review Board (IRB) protocols in accordance with the regulations of the Research Compliance Office at Stanford University (IRB# 18981).

### METHOD DETAILS

#### Single-cell RNA sequencing and data processing

Each sample was received directly from surgeons and promptly delivered to the laboratory on ice. Upon arrival at the laboratory, the samples were immediately processed. The ethmoid sinus mucosa was removed from the bone and nasal polyps were left intact and were minced into small pieces by scissors on ice. The minced tissues were placed into a C tube (Miltenyi Biotec, Bergisch Gladbach, Germany) within a solution of RPMI 1640 (Gibco, Grand Island, NY) containing 10% fetal bovine serum (FBS), 0.02 mg/ml DNase I (Millipore Sigma, St. Louis, MO), and 4 mg/ml collagenase type IV (Thermo Fisher Scientific). The mixture was homogenized using the gentleMACS Dissociator (Miltenyi Biotec) and incubated at 37°C for total of 30 minutes (15 minutes, 2 times) with rotated using MACSmix Tube Rotator (Miltenyi Biotec). Between and after the two incubations, they were also homogenized in a gentleMACS Dissociator. Finally, the samples were filtered through a 70-m cell strainer and spun down at 500g for 5 min. Red blood cells (RBC) were lysed using the RBC Lysis Solution (BioLegend, San Diego, CA) for 4 min at room temperature. Cells were then washed with ice-cold PBS and spun down at 500g for 5 min at 4°C before resuspension in RPMI containing 10% FBS. The cell suspension was filtered through 40-m Flowmi Cell Strainers (Millipore Sigma) and counted to adjust concentration to 1,000 cells per l. In all samples, the process up to this point was completed within 3 hours to minimize cell dissociation artifacts.

The single cell suspension was loaded onto the Chromium Controller (10x Genomics) using the Chromium single cell 3’ Reagent Kit v3 (10X Genomics), and scRNA-seq libraries generated in accordance with the manufacturer’s protocols. Sequencing was performed on a Illumina HiSeq 4000 with 75 bp pair end reads.

The CellRanger v3.1.0 (10X Genomics) analysis pipeline was used to generate a final digital expression matrix. Raw sequence reads were preprocessed and mapped onto the reference human genome (GRCh38-3.0.0). These data were used as input into the Seurat package (4.1.1) (https://github.com/satijalab/seurat) for further analyses in R (4.2.0). As part of the quality control metrics, genes detected (UMI count > 0) in less than three cells, and cells containing a small number of genes detected (UMI count < 200) or high mitochondrial genome transcript ratio (25%) were removed.

After normalizing and identifying variable features for each sample independently, the data from all the individuals were combined using the top 30 dimensions in ‘FindIntegrationAnchors()’ and ‘IntegrateData()’ functions.

#### Unsupervised clustering and cell type identification

The normalized expression level was calculated for each gene by dividing the read counts for each cell by the total counts and multiplied by a scale factor of 1,000,000. The natural-log transformed values were taken as the final measurement of expression level for each gene in a specific cell. Based on the normalized expression level, we next selected a subset of genes that with high cell-to-cell variation in the dataset. Then, the principal component analysis (PCA) was performed on these variable genes. Following the results of PCA, Harmony was performed to correct the batch effect among samples,^32^ then an adequate number (30-40) determined by Elbowplot of principal components (PCs) were selected for dimensionality reduction and clustering. The UMap algorithm with a resolution parameter in a range of 0.1-0.8 was applied for dimensionality reduction and visualization.^43^ To identify marker genes that define a cluster, differential expression analysis was performed by comparing each single cluster to all other cells. To accelerate the computational time of differential expression analysis, genes with > 0.25 logfold difference on average between the two groups of cells and detectable in more than 25% of cells in either of the two groups of cells were retained. Using the above differentially expressed genes, cells were annotated to different cell types according to their well-known canonical markers. All the above analysis was performed using the Seurat R package (v 4.1.1).^31^

#### Differentially expressed genes analysis in scRNA-seq data

To define genes that may function in between CRS with and without nasal polyps, differential expression analysis in specific cell groups was performed using the ‘FindMarkers’ function implemented in the Seurat package. The Wilcoxon rank sum test with log-scaled fold change > 0.25 and adjusted P value < 0.05 (bonferroni correction) was performed to select differentially expressed genes.

#### Pathway analysis

To reveal the potential biological functions of T cells in two types of CRS, GSEA was performed with R package ‘clusterProfiler’ and ‘ReactomePA’ to identify pathway enriched under the REACTOME gene sets released by MsigDB.^33,34,44,45^ In Tuft cells, differentially expressed genes identified between CRS with and without nasal polyps were used to perform WikiPathway enrichment.^46^ Pathways that have a BH-adjusted *P* value smaller than 0.05 were defined as being significantly enriched, and GSEA was performed to further validate the pathway enrichment.

#### Definition and calculation of gene signature scores

To assess the functional status of specific cells, relative signatures were collected from published literature as follows. A M2-like signature was used to define the functional phenotype of macrophages. An inflammatory signature,^46^ Th1 and Th2 signature^47,48^ were used to assess T cell functions. In scRNA data, expression scores of specific signatures were calculated using AddModuleScore in the Seurat package. To validate the interaction between basal cells and T2 immune response, the expression score and enrichment of cell fate signatures were accessed in public single cell and bulk RNA-seq datasets.^10^ All genes associated with each pathway score are available in Table S3. Violinplot was adopted to present the scoring difference among different types of CRS and healthy control samples, and Wilcoxon rank-sum test was performed to indicate the statistical significance.

#### Construction of cell developmental trajectory

The developmental trajectory of the basal cells was inferred using the Monocle2 package.^35^ The 10x Genomics sequencing data was first imported into Monocle2 in CellDataSet class, and the negative binomial distribution was chosen to model the reads count data. Differentially expressed genes across different cell populations were identified and selected as input features to construct the trajectory. Then, a Reversed Graph Embedding algorithm was performed to reduce the data’s dimensionality. With the expression data projected into a lower dimensional space, cells were ordered in pseudotime and trajectory was built to describe how cells transit from one state into another. After the cell trajectories were constructed, differentially expressed genes along the pseudotime trajectory separated by the branch point were detected using the ‘differentialGeneTest’ function. For each interested gene, the expression trend along the pseudotime was estimated using non-linear regression, and plotted with a curve chart.

To perform *in silico* TF perturbation experiments regarding the developmental trajectories of Basal cells, we implemented the python package CellOracle^26^ on our scRNA-seq data. In brief, we loaded the raw gene counts and the Monocle2 trajectory embedding (calculated as described in the previous paragraph) as the input for CellOracle object. We utilized the prebuilt promoter base-GRN "hg19_gimmemotifsv5_fpr2" in our pipeline for simulation, and all other parameters were set as default, same as described in the official documentation files. In our specific analysis, we tested KLF4 and KLF6, which are two TFs where we observed significant change in their gene expression levels in our DEG analysis. For visualization, we generated the plots via function "plot_dev_flow_on_grid" and function "plot_inner_product_on_grid".

#### Definition of Basal Transition Continuum

To define the basal transition continuum and identify differentially infiltrated immune cells, we compared gene expression profiles of basal cells from each CRS to basal cells from control samples. Only samples with more than 50 basal cells were included to enhance the statistical reliability. Differential gene expression was computed using the Seurat function FindMarkers, with the following parameters: min.pct=0, logfc.threshold=0, min.cells.feature=0, max.cells.per.ident=300, and using Model-based Analysis of Single-cell Transcriptomics (MAST) as the differential testing method. Genes were considered differentially expressed if they met the criteria of an adjusted p-value (Padj) ≤ 0.05, ∣log2FC∣ ≥ 0.5 in at least two samples. The differential genes identified were used to perform principal component analysis, then a spline was fit to the first two principal components by loess regression to construct the basal transition continuum.

#### Inference of cell-cell communications

R package Cellchat (v1.5.0) was adopted to identify significant ligand-receptor pairs within different types of CRS samples in scRNA-seq.^36^ Ligand-receptor communication probabilities/strengths were computed, tested, compared and visualized on the samples of CRS with and without nasal polyps. The minimum communication cells threshold was set to 10 and other parameters were left as default.

#### GeoMx-Digital Spatial Profiling

Samples collected for NanoString GeoMx-Digital Spatial Profiling were fixed in 10% neutral buffered formalin at room temperature for 24-48 hours at a volume 10 times greater than the tissue volume. The specimens were then washed in 1xPBS, dehydrated, and paraffin-embedded at the Stanford Histology Core, and sectioned onto Fisher Superfrost Plus microscope slides (Fisher, 12-500-15) at 5um.

Slides were deparaffinized and prepared according to the official NanoString GeoMx-NGS RNA Manual Slide Preparation protocol.^49^ In brief, slides were baked for 30 min at 60°C before washing in Xylene (3 x washes at 5 min each), 100% EtOH (2 x washes at 5 min each), 95% EtOH (1 x wash at 5 min) and in 1X PBS (1 x wash at 1 min). Slides then underwent heat induced epitope retrieval at 99°C for 10 min in Tris-EDTA retrieval buffer (eBioscience, 00-4956-58).

Slides were then digested by Protease K (0.1g/ml) for 5 mins at 37°C, and then washed with 1X PBS. Subsequently, slides were fixed by 10% neutral buffered formalin (EMS Diasum, 15740-04) for 5 min at room temperature, then the fixation process was stopped by 5 mins of 1X NBF Stop Buffer wash, followed by 5 mins of 1X PBS wash. The NanoString DSP Human CTA detection probe cocktail was then applied to the slides and incubated overnight (~18 hrs) at 37°C. After hybridization, slides were washed in Stringent Wash Buffer (2X SSC, 50% Formamide) 2 times, every 5 mins. Slides were then washed by 2X SSC twice, 2 mins each. Buffer W was then applied to the slides for 30 mins, followed by antibody staining for 1hr: CD45 (D9M8I, Cell Signaling Technologies), PanCK (AE1 + AE3, Novus). Slides were then washed by 2X SSC twice, 5mins each, and stained with 500nM SYTO 13 for 15 min, then loaded on the GeoMx instrument. For GeoMx DSP sample collection, we followed the instructions described in the GeoMx DSP instrument user manual (MAN-10088-03). For the single-cell RNA sequencing validation cohort, individual ROIs were selected in the areas of immune cell aggregation and where epithelium were presented on the apical side of tissues. The CD45 positive or PanCK positive masks were chosen with the agreement of two or more investigators. On average, the ROI sizes are approximately 45217 um2 for CD45^+^ regions and 37501 um2 for PanCK^+^ regions. For the large-scale validation cohort, we followed a previously established protocol.^27^ Briefly, cell segmentation of the GeoMx datasets was conducted using a local implementation of deep-cell-tf, with the SYTO13 channel used for the nucleus and the summation of CD45, CD68, and PanCK as the membrane feature. Cell phenotyping was performed using PhenoGraph (k = 200), leveraging CD45, CD68, and PanCK to identify immune cells, myeloid cells, and epithelial cells.^37^ Annotations were verified using Mantis Viewer,^38^ and binary cell masks corresponding to these three cell phenotypes were generated for each ROI to enable downstream transcript extraction. On average, the ROI sizes were approximately 59,223 μm^2^ for immune cell regions, 80,727 μm^2^ for myeloid cell regions, and 101,826 μm^2^ for epithelium regions. After sample collection, the NanoString NGS library preparation kit was used: Each ROI was uniquely indexed using Illumina’s i5 x i7 dual-indexing system. In total, 4 μL of collected samples was used in a PCR reaction with 1 μM of i5 primer, 1 μM i7 primer, and 1 x NanoString library prep PCR Master Mix. PCR reaction conditions were 37°C for 30 min, 50 °C for 10 min, 95°C for 3 min, 18 cycles of 95°C for 15 s, 65 °C for 60 s, 68°C for 30s, and final extension of 68°C for 5min. Then the product was purified with two rounds of AMPure XP beads (Beckman Coulter) at 1.2 x bead-to-sample ratio. Libraries were paired-end sequenced (2 × 75) on a NextSeq550.

#### Digital Spatial Profiling Data Analysis

Probes from the NanoString CTA panel were mapped and counted using the NanoString GeoMx Data Analysis software pipeline,^49^ using the FASTQ output from NGS sequencing. For the single-cell RNA sequencing validation cohort, the data underwent quality control and normalization steps with the ‘Geomx-Tools’ software from NanoString: First, ROI and probes that did not meet the default QC requirement were filtered out and not used in the subsequent analysis. Next, raw probe counts were transferred into a gene-level count matrix by calculating the geometric mean of probes for each gene. Normalization of gene counts were then performed, with the ‘Q3 norm’ method in ‘Geomx-Tools’. The Q3 normalized gene counts were then used for all subsequent downstream analysis. For the large-scale validation cohort using the NanoString WTA panel, we extended our previously established pipeline^27^ based on the standR workflow.^39^ This included normalization, negative control genes (NCGs) identification, and batch effect correction using the RUV4 method. After a comprehensive grid search, log counts-per-million reads (CPM) were selected for normalization, with 1500 NCGs and six k-covariance matrices set for RUV4. This batch-corrected data was used for downstream analyses.

Mean levels of spatial region-specific gene expression or mean levels of spatial expression scores of specific signatures, and their correlations were adopted to validate corresponding results or hypotheses. Apart from published signatures, differential expressed genes identified in scRNA data were also applied to validate cell phenotype and function in the DSP data, and spatial region-specific expression scores were calculated with ssGSEA using the GSVA package.^40^ The Wilcoxon rank sum test was performed to calculate the significance of differences between samples.

#### cNMF and Co-expressed Gene Program Analysis

To perform *de novo* gene program identification, python package "cNMF" was utilized, as previously described by Kotliar et al.^41^ The rank in cNMF (number of gene programs) was set to 35 (determined via the function "k_selection_plot"). After identifying the gene programs, the Spearman correlation of the program scores between paired CD45 and PanCK regions was calculated, and plotted as a heatmap with programs clustered by hierarchical clustering. To annotate the "correlation hotspots" of gene programs, we first selected the top 20 genes for each gene program, based on ranking from "gep_scores", combine them across all the gene programs in the same "correlation hotspot", then we utilized the function "enrichr" in the R package "enrichR", with the database "GO_Biological_Process_2015", on these pooled genes and annotated the gene program "hotspots".

#### Calculation of geometric mean of EPI-IMM interaction odds ratios

To assess the relative likelihood of across-type (EPI-IMM) interactions compared to within-type interactions (EPI-EPI and IMM-IMM), the odds for each interaction type were calculated. Let $e_{AB}$, $e_{AA}$, and $e_{BB}$ represent the observed number of across-type, EPI-EPI, and IMM-IMM connections, respectively, while $n_{A}$ and $n_{B}$ are the numbers of epithelial and immune cells. The total possible across-type connections ($N_{AB}$) were calculated as $n_{A}\times n_{B}$, and within-type connections ($N_{AA}$ and $N_{BB}$) as

$$N_{AA}=\frac{n_{A}\times (n_{A}-1)}{2}\;\text{and}\;N_{BB}=\frac{n_{B}\times (n_{B}-1)}{2},$$

respectively. The odds for across-type interactions (${\text{Odds}}_{AB}$) were computed as the ratio of observed connections to the remaining possible connections,

$${\text{Odds}}_{AB}=\frac{e_{AB}}{N_{AB}-e_{AB}},$$

and similarly for within-type odds (${\text{Odds}}_{AA}$ and ${\text{Odds}}_{BB}$). To compare across-type interactions with within-type interactions, odds ratios were computed as:

$${\text{OR}}_{AB∕AA}=\frac{{\text{Odds}}_{AB}}{{\text{Odds}}_{AA}}\;\text{and}\;{\text{OR}}_{AB∕BB}=\frac{{\text{Odds}}_{AB}}{{\text{Odds}}_{BB}}$$

providing a measure of the relative likelihood of across-type interactions against within-type interactions.

To compare the odds of across-type interactions with the combined odds of within-type interactions, we used the geometric mean of the odds for within-type interactions. The geometric odds ratio, denoted as $OR^{*}$, was computed as follows:

$$OR\;*=\sqrt{\frac{{\text{Odds}}_{AB}}{{\text{Odds}}_{AA}}\times \frac{{\text{Odds}}_{AB}}{{\text{Odds}}_{BB}}}$$

To investigate the ability of IMM recruitment from EPI, we analyzed the co-expression of specific ligand-receptor pairs that drive IMM recruitment from EPI. The ligand-receptor pairs, including *CCL26-CCR3*,^50^ *CCL20-CCR6*,^51^ *TSLP-IL7R*,^52^ and *IL33-IL1RL1*^53^ from the previous analysis, were selected. For each ROI, expression levels of ligands and receptors were extracted, and an interaction score was calculated by multiplying the ligand expression in EPI with the receptor expression in IMM within the same ROI. The interaction score for each ligand-receptor pair was calculated as the ratio of the ligand-receptor co-expression to the geometric odds ratio, providing a weighted co-expression score:

$$P_{i,j}=\frac{L_{i,j}R_{i,j}}{{OR}_{j}^{*}}$$

where $L_{i,j}$ is the expression value of the ligand in EPI cells within the jth ROI, $R_{i,j}$ is the expression value of the corresponding receptor in IMM cells within the jth ROI, and $OR^{*}$ is the geometric odds ratio for across-type interaction (EPI-IMM), calculated as previously described. To derive an overall measure of EPI-IMM interactions driven by selected ligand-receptor pairs, the geometric mean of weighted co-expression scores was calculated and provided a balanced representation of interaction strength across multiple ligand-receptor pairs:

$${\text{Interaction Score}}_{j}={\left(\prod_{i=1}^{n}P_{i,j}\right)}^{\frac{1}{n}}$$

where n is the number of ligand-receptor pairs under consideration (*n* = 4 in this study), and $P_{i,j}$ represents the weighted co-expression score for the ith ligand-receptor pair in the jth ROI.

#### Hematoxylin and Eosin Staining and Eosinophil Infiltration Scoring

Tissue sections serial to those used in GeoMX profiling were deparaffinized by incubation in xylene and rehydrated through a graded ethanol series to water. Staining was performed using hematoxylin and eosin staining solutions (Biosharp). Sections were stained with hematoxylin for 50 seconds, followed by a quick rinse in water. The sections were then briefly dipped in 1% acid alcohol for 1 second, rinsed again in water, and immersed in running water for 30 minutes to allow for bluing. Staining progress was monitored under a microscope. Next, the sections were stained in eosin solution for 3 minutes to highlight tissue structures outside of the nuclei. Following eosin staining, sections were rinsed in water for 5 seconds to remove excess dye. Sections were then sequentially dehydrated in 80% ethanol for 10 seconds, followed by two rounds of 95% ethanol for 2 minutes each. Finally, the sections were dehydrated in absolute ethanol for 2 minutes, repeated twice, and cleared in xylene for 5 minutes, also repeated twice. The processed slides were air-dried in a fume hood and mounted using neutral resin.

Eosinophil infiltration scoring was determined by quantifying the number of eosinophils located in or near epithelium regions per high-power field (HPF, 200× magnification) by a board-certified pathologist (D.N.). For each sample, 3 to 5 fields with the highest levels of infiltration were selected and analyzed, and the mean score of these fields was recorded as the final score.

### QUANTIFICATION AND STATISTICAL ANALYSIS

All data analyses were conducted in R 4.2.0. Statistical significance was defined as a twosided *P* value of less than 0.05, with tests as specified below. The comparison of cell fractions, expression levels of marker genes and gene signature scores among different types of CRS and control samples were performed using the Wilcoxon rank sum test. The correlation analyses were performed using the Spearman’s correlation test.

### ADDITIONAL RESOURCES

A data portal^30^ was developed to facilitate convenient access and visualization of all data associated with this study: https://bmblx.bmi.osumc.edu/immune_epi/.

## Supplementary Material

S1 Figures

S1 Table

S2 Table

Supplemental information can be found online at https://doi.org/10.1016/j.immuni.2025.08.009.

## Figures and Tables

**Table:** KEY RESOURCES TABLE

REAGENT or RESOURCE	SOURCE	IDENTIFIER
Antibodies		
CD45 (Intracellular Domain) (D9M8I) XP^®^ Rabbit mAb (BSA and Azide Free)	Cell Signaling Technologies	Cat#47937
Cytokeratin, pan Antibody (AE-1/AE-3) - Azide and BSA Free	Novus	Cat#NBP2-33200
CD68 (D4B9C) XP^®^ Rabbit mAb (BSA and Azide Free) #26042	Cell Signaling Technologies	Cat#26042
Alexa Fluor^®^ 532 Antibody Labeling Kits	Life Technologies	Cat#A20182
Alexa Fluor^®^ 594 Antibody Labeling Kits	Life Technologies	Cat#A20185
Alexa Fluor^®^ 647 Antibody Labeling Kits	Life Technologies	Cat#A20186
GeoMx Solid Tumor TME Morphology Kit	Nanostring	Cat#GMX-RNA-MORPH-HST-12
Biological samples		
Human ethmoid sinus mucosa and nasal polyp samples	Stanford University, Dokkyo University and National Taiwan University	N/A
Chemicals, peptides, and recombinant proteins		
Formalin, Neutral, Buffered 10% w/v in Phosphate Buffer	Electron Microscopy Sciences	Cat#15740-04
SSC Buffer 20× Concentrate	Millipore Sigma	Cat#S6639-1L
Critical commercial assays		
Chromium Next GEM Single Cell 3′ Kit v3.1	10x Genomics	Cat#PN-1000121
GeoMx Human Whole Transcriptome Atlas	Nanostring	Cat#121401102
GeoMx Seq Code Pack: A, B, C, & D	Nanostring	Cat#121400205
GeoMx RNA Slide Prep Kit	Nanostring	Cat#121300313
GeoMx DSP Instrument Buffer Kit	Nanostring	Cat#100474
GeoMx DSP Collection Plate	Nanostring	Cat#100473
IHC Antigen Retrieval Solution – High pH (10X)	eBioscience	Cat#00-4956-58
AMPure XP beads, 5 mL	Beckman Coulter	Cat#A63880
Deposited data		
CRS Digital Spatial Profiling datasets	This paper	https://doi.org/10.5281/zenodo.13947018
CRS DSP and scRNA-seq datasets	This paper	CRS data portal: https://bmblx.bmi.osumc.edu/immune_epi/
scRNA-seq dataset of In vivo blockade treated CRS patients	Shalek Laboratory	Ordovas-Montanes et al.^10^
Bulk RNA-seq dataset of cytokine stimulated basal cell	Shalek Laboratory	Ordovas-Montanes et al.^10^
CRS scRNA-seq dataset	Wang et al.^17^	GSA (Genome Sequence Archive) HRA000772
Software and algorithms		
CellRanger v3.1.0	10x Genomics	https://support.10xgenomics.com/single-cell-gene-expression/software/pipelines/3.1/installation
Seurat v4.1.1	Hao et al.^31^	https://github.com/satijalab/seurat/releases/tag/v4.1.1
R v4.2.0	R Core	https://www.r-project.org/
Harmony v0.1.0	Korsunsky et al.^32^	https://github.com/immunogenomics/harmony
clusterProfiler v4.0.0	Wu et al.^33^	https://guangchuangyu.github.io/software/clusterProfiler
ReactomePA v1.26.0	Yu et al.^34^	https://guangchuangyu.github.io/software/ReactomePA/
Monocle v2.36.0	Trapnell et al.^35^	https://cole-trapnell-lab.github.io/monocle-release/
Cellchat v1.5.0	Jin et al.^36^	https://github.com/sqjin/CellChat
PhenoGraph v1.5.7	Levine et al.^37^	https://github.com/dpeerlab/phenograph
Mantis Viewer v1.2.0	Schiemann et al.^38^	https://github.com/CANDELbio/mantis-viewer
standR v1.12.0	Liu et al.^39^	https://github.com/DavisLaboratory/standR
GSVA v2.2.0	Hänzelmann et al.^40^	https://github.com/rcastelo/GSVA
cNMF v1.6	Kotliar et al.^41^	https://github.com/dylkot/cNMF
CellOracle v0.17.1	Kamimoto et al.^26^	https://github.com/morris-lab/CellOracle
Python	Python Software Foundation	https://www.python.org

## Highlights

- Multi-scale single-cell and spatial atlas of CRS with and without NPs
- Detailed mapping of immune-epithelial interactions in inflamed nasal tissue
- Basal progenitor and tuft cell dysregulation drives epithelial remodeling
- Basal cell trajectory reveals link between cell fate and NP formation
