# Supplementary material for: Multi-scaled transcriptomics of chronically inflamed nasal epithelium reveals immune-epithelial dynamics and tissue remodeling in nasal polyp formation
Source: Immunity. Author manuscript; Available in PMC 2026 Jan 30. (PMC12857809; doi:10.1016/j.immuni.2025.08.009)
Supplement: S1 Figures [file NIHMS2134457-supplement-S1_Figures.pdf]

Immunity, Volume *58*

Supplemental information

Multi-scaled transcriptomics of chronically inflamed nasal epithelium reveals immune-epithelial dynamics and tissue remodeling in nasal polyp formation

Guanrui Liao, Tsuguhisa Nakayama, Bokai Zhu, Ivan T. Lee, Jason Yeung, Yao Yu Yeo, Yuzhou Chang, Cankun Wang, Steven Chun-Kang Liao, Dingani Nkosi, Axel Renteria, Dawn T. Bravo, Jonathan B. Overdevest, Carol H. Yan, David Zarabanda, Philip A. Gall, Sachi S. Dholakia, Nicole A. Borchard, Angela Yang, Dayoung Kim, Zara M. Patel, Peter H. Hwang, Dhananjay Wagh, John Coller, Katie M. Phillips, Michael T. Chang, Matt Lechner, Zihai Li, Te-Huei Yeh, Garry Nolan, Maria Serena Longhi, Vassiliki Boussiotis, Dan H. Barouch, Qin Ma, Jayakar V. Nayak, and Sizun Jiang

Supplementary Figure Legends

Figure S1. Comprehensive Single-Cell Transcriptomic Analysis Reveals the Immune Microenvironment in CRS, related to Figure 1. (A) Violin plots showing number of unique genes (left), number of total molecules (middle) and percentage of mitochondrial counts (right) of each cell in the single cell dataset. (B) UMAP plots showing the distribution of immune cells, colored by their origins, with the left panel representing tissue types and the right panel representing patient samples. Adjacent bar plots illustrating the composition of each cell type by tissue type (left) and by patient sample (right). (C) UMAP plots showing the expression of selected marker genes for the defined immune cell groups. (D) Barplots depicting the comparison of other immune cell fractions with significant differences between CRS and control samples using the Wilcoxon test (two-sided).

Figure S2. Polarization of Macrophages to M2 Phenotype and Enrichment of Mast Cell in Nasal Polyps Correlates with Type 2 Immune Responses, related to Figure 2. (A) UMAP plots showing the distribution of myeloid cells, colored by their origins, with the left panel representing tissue types and the right panel representing patient samples. Adjacent bar plots illustrating the composition of each cell type by tissue type (left) and by patient sample (right). (B) Violin plot illustrating M1 scores for macrophages across CRS and control samples, with comparisons performed using the Wilcoxon test (two-sided) and p values indicated. (C) Violin plots validating M1 and M2 scores for macrophages across CRS and control samples from public dataset, with comparisons performed using the Wilcoxon test (two-sided) and p values indicated. (D) Violin plot comparing expression scores of M2 signature between CRS nasal polyps (purple) and healthy control samples (red) in GeoMx data within CD45+ regions. (eCRSwNP: eosinophilic CRSwNP) (E) Violin plots validating expression levels of *CCL13* and *CCL18* in macrophages across CRS and control samples from (Wang, 2022)[17], with comparisons performed using the Wilcoxon test (two-sided) and p values indicated. (F) Heatmap illustrating the normalized expression of *CCL13* and *CCL18* across different immune cell clusters in scRNA-seq data. (G) Violin plots comparing eosinophil spatial signature expression scores between CRS nasal polyps (purple) and healthy control samples (orange) in spatial transcriptomics GeoMx data within CD45+ regions (left panel) and PanCK+ regions (right panel), with comparisons performed using the Wilcoxon test (two-sided) and p values indicated. (H) Scatter plots demonstrating the correlation between *CCL18* mRNA expression levels in situ, and eosinophil spatial signature expression scores in GeoMx data, with data origins colored to indicate CD45+ regions (magenta) and PanCK+ regions (yellow). The data was fitted using a linear regression model, with blue lines indicating the mean and grey regions highlighting the 95\% confidence intervals. The regression index and p values are provided within the plots. (I) Scatter plots illustrating the correlation between *CCL18* expression levels and eosinophil signature scores in CD45+ or PanCK+ regions of GeoMx data, with sample origins color-coded to represent CRS nasal polyps

(purple) and healthy control samples (orange). Diagrams above the scatter plots indicate regions where *CCL18* and eosinophil spatial gene signatures were captured. (J) Stacked barplot illustrating the proportion of cores with varying eosinophil infiltration scores in epithelial regions, comparing CRS and control samples. The enrichment of samples with high infiltration scores in CRSwNP was evaluated using a chi-square test. (K) Scatter plots depicting *IL4* and *IL13* expression levels in various immune cells, and their dominant expression in Mast cells.

Figure S3. Tuft Cells in Nasal Polyps Correlate with Th2 Cells, related to Figure 3. (A) UMAP plots showing the distribution of epithelial cells, colored by their origins, with the left panel representing tissue types and the right panel representing patient samples. Adjacent bar plots illustrating the composition of each cell type by tissue type (left) and by patient sample (right). (B) UMAP plots showing the expression of selected marker genes for the defined epithelial cell groups. (C) Barplots depicting the comparison of other epithelial cell subtype fractions between CRS and control samples. (D) Enrichment plot of the prostaglandin synthesis and regulation pathway in Tuft cells from CRSwNP versus CRSsNP, using GSEA analysis with the WIKIPATHWAY gene set. The enrichment score and p-value are indicated in the plot. (E) Barplot validating the enrichment of Tuft cells in CRSwNP. (F) Enrichment plot validating the enrichment of prostaglandin synthesis and regulation pathway in Tuft cells from CRSwNP versus CRSsNP from public dataset, using GSEA analysis with the WIKIPATHWAY gene set. The enrichment score and p-value are indicated in the plot.

Figure S4. Nascent Basal Cells in Nasal Polyps Exhibit a Unique Transition Trajectory and Induce T2 Immune Response, related to Figure 4. (A) Volcano plot depicting differentially expressed genes in cycling basal cells between CRS nasal polyps and CRS without nasal polyps. The most significant genes are highlighted in red (|Fold change| > 1.5). (B) Venn plot depicting overlap between upregulated genes in suprabasal cells and cycling cells in nasal polyps. (C) Dynamic expression score of functional pathway signatures upregulated in CRS without nasal polyps during basal cell transition along pseudotime in CRS nasal polyps (purple) and CRS without nasal polyps (green). (D) Scatter plot and regression line illustrating the correlation between Cell-fate2 basal cell spatial gene signature expression scores in PanCK+ regions and eosinophil cell spatial gene signature expression scores in CD45+ regions. Dots are colored to represent patient sample origins. The grey region indicates the confidence interval. The regression index and p-values are shown in the plots. (E) Violin plots validating expression scores of chronic inflammation and immune remodeling signatures for basal cells across CRS and control samples from (Wang, 2022)[17], with comparisons performed using the Wilcoxon test (two-sided) and p values indicated. (F) Dynamic expression of transcription factor genes differentially expressed in CRS without nasal polyps (green) and CRS nasal polyps (purple) during basal cell transition along pseudotime. (G) The pseudotime trajectory following *KLF4* knockout simulations was computed and converted into 2D pseudotime gradient vectors to illustrate cell state transitions. (H) Perturbation scores for the *KLF4* knockout simulations were

calculated based on changes in vector direction post-knockout compared to the original vector direction. A negative score (indicated in purple) suggested that the transcription factor knockout delayed or inhibited differentiation, while a positive score (indicated in green) suggested an enhancement or promotion of differentiation. (I) Enrichment plot validating the enrichment of pathways identified to be upregulated in immune remodeling cell fate using GSEA analysis in basal cells from public dataset. The enrichment score and p-value are indicated in the plot. (J) Violin plots comparing the expression level of *KLF4* between basal cells in pre-treatment (blue) and post-treatment (red) nasal polyps samples from an individual treated with IL-4Rα antibody. (K) Barplot showing the enrichment of basal cell-fate signature in IL4/13 cytokine stimulated Non-polyp derived basal cells compared with Non-polyp derived basal cell baseline.

Figure S5. Figure S5. Dynamics of immune cell representation in basal transition, related to Figure 4. (A) Basal transition continuum based on PCA analysis. Principal components were computed on the log2FC values between basal cells from each CRS sample and healthy control samples for the set of peaks and genes that were significantly differential (Wilcoxon FDR $\leq$ 0.05 and |log2FC | $\geq$ 0.5 for peaks; MAST test for genes). A spline was fit to the first two principal components and samples were ordered based on their position along the spline. Samples are colored based on the origin of tissue types. (B) Proportion of cell types in immune cells in each scRNA-seq sample plotted against position of the sample in the basal transition continuum. Samples are colored based on the origin of tissue types.

Figure S6. Spatial Analysis Confirms the Immune Remodeling Effect of Epithelial Cells in Nasal Polyps, related to Figure5 & 6. (A, B) Principal component analysis of all ROIs in prior (A) and post (B) batch-effect correction dataset from the cluster-based annotated GeoMX cohort. The first two principal components and their eigenvalues are shown. ROIs are colored based on the origin of GeoMX slide, annotated segment label and tissue type. (C) Barplots comparing expression scores of Th1/2 signatures between CRS and healthy control samples within immune cell regions. (C) Barplots comparing expression scores of Th1/2 signatures between CRS and healthy control samples within immune cell regions using the Wilcoxon test (two-sided). (D) Barplot comparing expression scores of M2 signatures between CRS and healthy control samples within myeloid cell regions using the Wilcoxon test (two-sided). (E) Barplot comparing expression scores of eosinophil signatures between CRS and healthy control samples within myeloid cell regions using the Wilcoxon test (two-sided). (F) Scatter plots illustrating the correlation between \textit{CCL13} (upper panel) and \textit{CCL18} (bottom panel) expression levels and eosinophil signature scores in myeloid cell regions, with sample origins color-coded to represent CRS nasal polyps (purple), and CRS without nasal polyps (green) and healthy control samples (red). (G) Volcano plot depicting differentially expressed genes in spatial immune cell regions between CRS nasal polyps and CRS without nasal polyps. The most significant genes

are highlighted in red (|Fold change| > 1.5). (H) Pathways enriched in spatial immune cell regions from CRSwNP and CRSsNP, based on REACTOME enrichment analysis.

FigureS1

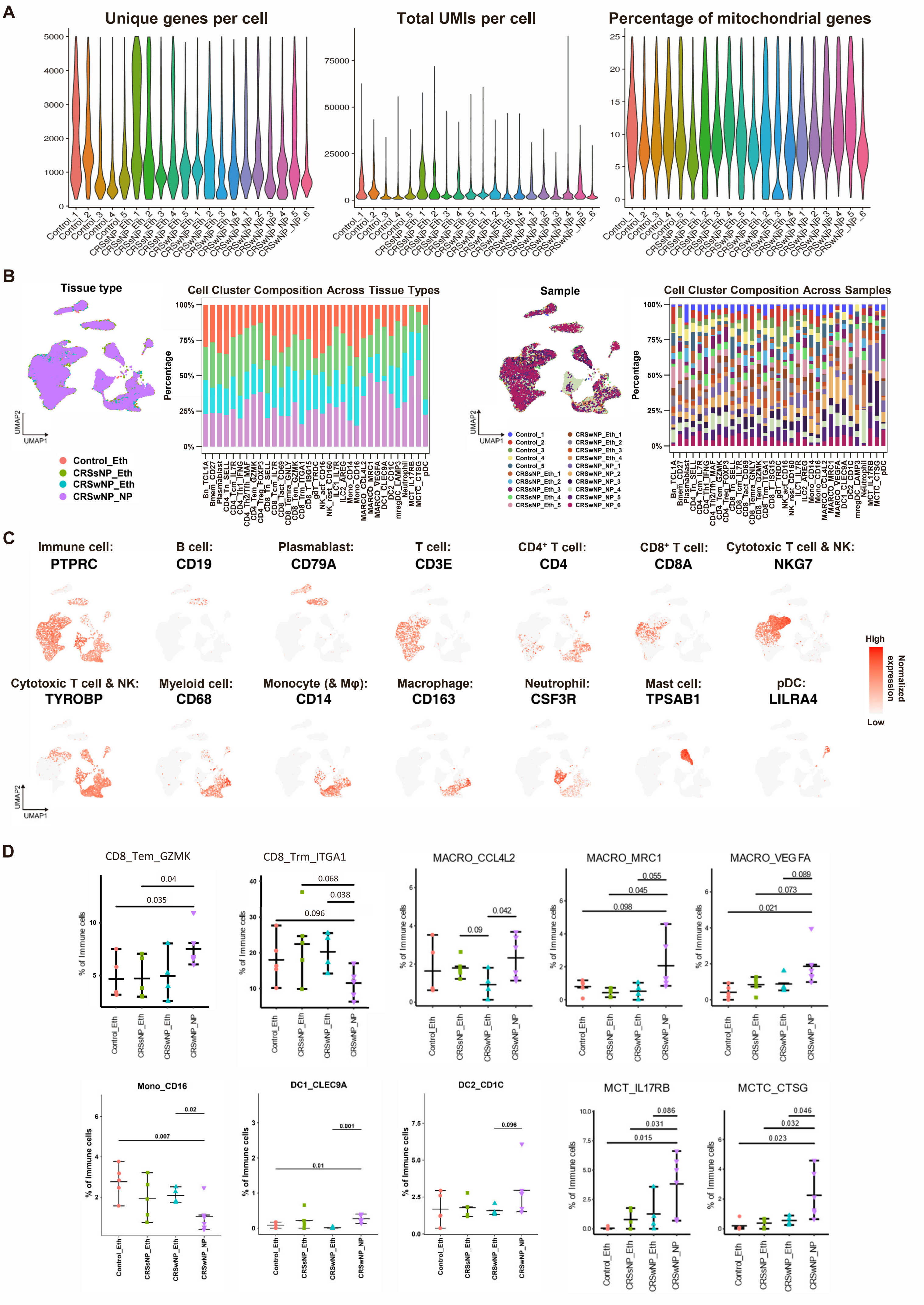
A
Unique genes per cell
Total UMIs per cell
Percentage of mitochondrial genes
B
Tissue type
Cell Cluster Composition Across Tissue Types
Sample
Cell Cluster Composition Across Samples
Percentage
UMAP1
UMAP2
Control_Eth
CRSsNP_Eth
CRSwNP_Eth
CRSwNP_NP
C
Immune cell: PTPRC
B cell: CD19
Plasmablast: CD79A
T cell: CD3E
CD4⁺ T cell: CD4
CD8⁺ T cell: CD8A
Cytotoxic T cell & NK: NKG7
Cytotoxic T cell & NK: TYROBP
Myeloid cell: CD68
Monocyte (& Mφ): CD14
Macrophage: CD163
Neutrophil: CSF3R
Mast cell: TPSAB1
pDC: LILRA4
High
Low
Normalized expression
D
CD8_Tem_GZMK
CD8_Trm_ITGA1
MACRO_CCL4L2
MACRO_MRC1
MACRO_VEGFA
Mono_CD16
DC1_CLEC9A
DC2_CD1C
MCT_IL17RB
MCTC_CTSG
% of Immune cells

FigureS2

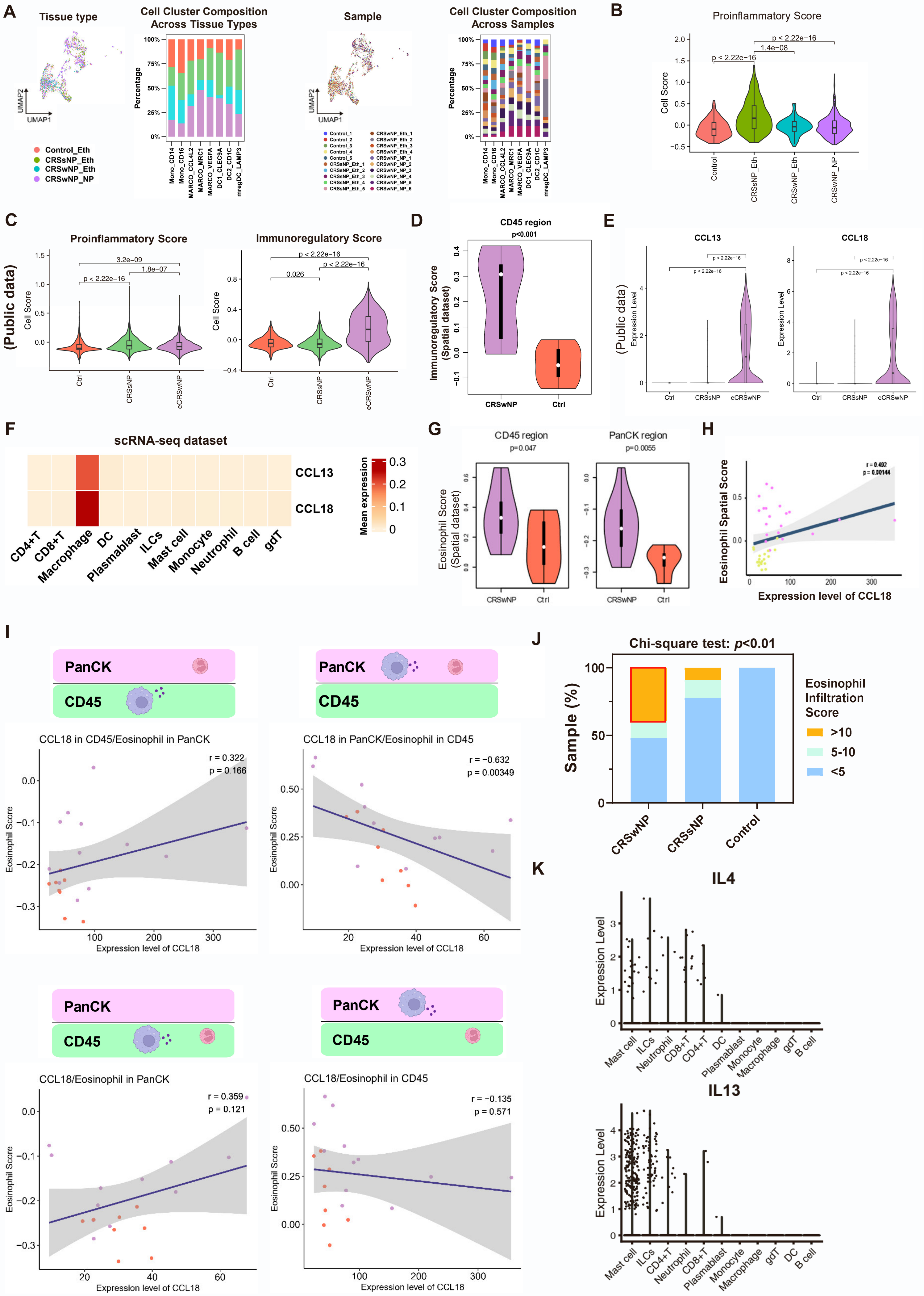
A
Tissue type
Cell Cluster Composition Across Tissue Types
Sample
Cell Cluster Composition Across Samples
Control_Eth
CRSsNP_Eth
CRSwNP_Eth
CRSwNP_NP
B
Proinflammatory Score
C
(Public data)
Proinflammatory Score
Immunoregulatory Score
D
CD45 region
p<0.001
Immunoregulatory Score (Spatial dataset)
E
CCL13
CCL18
F
scRNA-seq dataset
CCL13
CCL18
Mean expression
G
CD45 region
PanCK region
Eosinophil Score (Spatial dataset)
H
Eosinophil Spatial Score
Expression level of CCL18
I
PanCK
CD45
CCL18 in CD45/Eosinophil in PanCK
CCL18 in PanCK/Eosinophil in CD45
CCL18/Eosinophil in PanCK
CCL18/Eosinophil in CD45
Expression level of CCL18
Eosinophil Score
J
Chi-square test: p<0.01
Sample (%)
Eosinophil Infiltration Score
>10
5-10
<5
CRSwNP
CRSsNP
Control
K
IL4
IL13
Expression Level

FigureS3

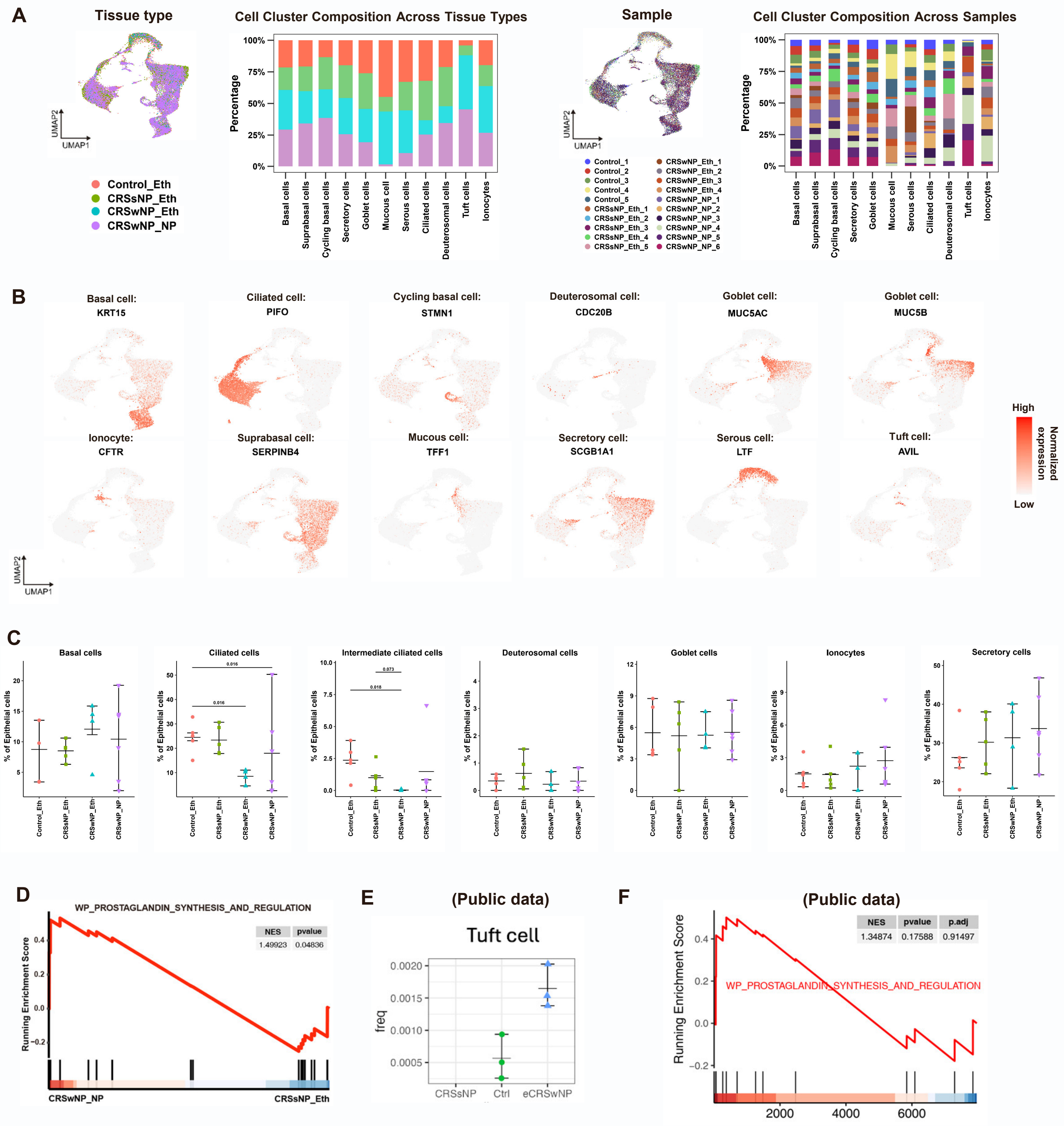

A
Tissue type
UMAP2
UMAP1
Control_Eth
CRSsNP_Eth
CRSwNP_Eth
CRSwNP_NP
Cell Cluster Composition Across Tissue Types
Percentage
100%
75%
50%
25%
0%
Basal cells
Suprabasal cells
Cycling basal cells
Secretory cells
Goblet cells
Mucous cell
Serous cells
Ciliated cells
Deuterosomal cells
Tuft cells
Ionocytes
Sample
Control_1
Control_2
Control_3
Control_4
Control_5
CRSsNP_Eth_1
CRSsNP_Eth_2
CRSsNP_Eth_3
CRSsNP_Eth_4
CRSsNP_Eth_5
CRSwNP_Eth_1
CRSwNP_Eth_2
CRSwNP_Eth_3
CRSwNP_Eth_4
CRSwNP_NP_1
CRSwNP_NP_2
CRSwNP_NP_3
CRSwNP_NP_4
CRSwNP_NP_5
CRSwNP_NP_6
Cell Cluster Composition Across Samples
B
Basal cell: KRT15
Ciliated cell: PIFO
Cycling basal cell: STMN1
Deuterosomal cell: CDC20B
Goblet cell: MUC5AC
Goblet cell: MUC5B
Ionocyte: CFTR
Suprabasal cell: SERPINB4
Mucous cell: TFF1
Secretory cell: SCGB1A1
Serous cell: LTF
Tuft cell: AVIL
High
Low
Normalized expression
C
Basal cells
Ciliated cells
Intermediate ciliated cells
Deuterosomal cells
Goblet cells
Ionocytes
Secretory cells
% of Epithelial cells
Control_Eth
CRSsNP_Eth
CRSwNP_Eth
CRSwNP_NP
0.016
0.016
0.073
0.018
D
WP_PROSTAGLANDIN_SYNTHESIS_AND_REGULATION
NES
pvalue
1.49923
0.04836
Running Enrichment Score
CRSwNP_NP
CRSsNP_Eth
E
(Public data)
Tuft cell
freq
0.0020
0.0015
0.0010
0.0005
CRSsNP
Ctrl
eCRSwNP
F
(Public data)
NES
pvalue
p.adj
1.34874
0.17588
0.91497
WP_PROSTAGLANDIN_SYNTHESIS_AND_REGULATION
Running Enrichment Score
2000
4000
6000

FigureS4

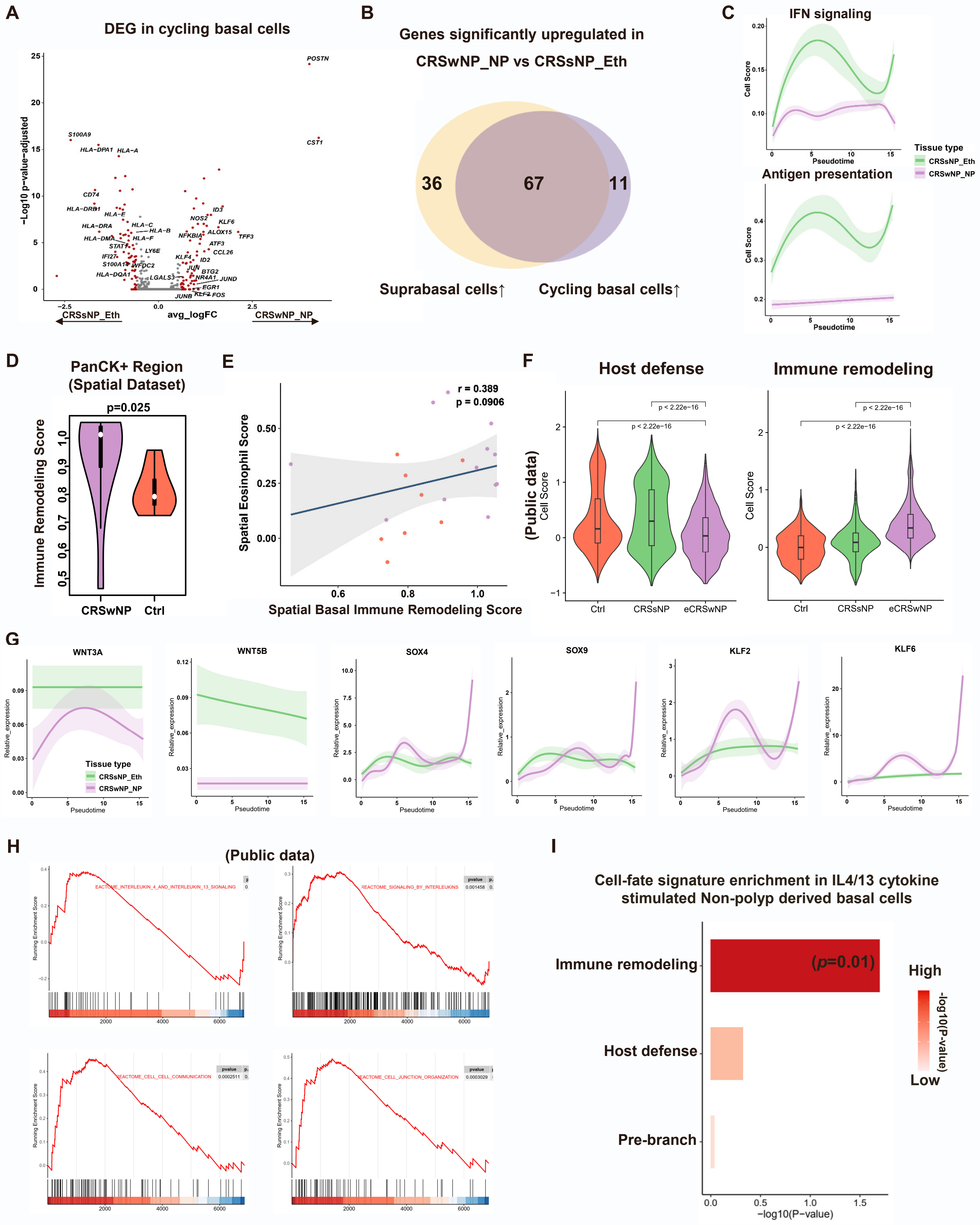
A
DEG in cycling basal cells
−Log10 p-value-adjusted
avg_logFC
CRSsNP_Eth
CRSwNP_NP
POSTN
CST1
S100A9
HLA-DPA1
HLA-A
CD74
HLA-DRB1
HLA-E
HLA-DRA
HLA-DMA
HLA-C
HLA-B
HLA-F
STAT1
LY6E
IFI27
S100A14
WFDC2
HLA-DQA1
LGALS3
NOS2
ID3
KLF6
NFKBIA
ALOX15
TFF3
ATF3
CCL26
KLF4
ID2
JUN
BTG2
NR4A1
JUND
EGR1
JUNB
KLF2
FOS
B
Genes significantly upregulated in
CRSwNP_NP vs CRSsNP_Eth
36
67
11
Suprabasal cells↑
Cycling basal cells↑
C
IFN signaling
Cell Score
Pseudotime
Tissue type
CRSsNP_Eth
CRSwNP_NP
Antigen presentation
D
PanCK+ Region
(Spatial Dataset)
p=0.025
Immune Remodeling Score
CRSwNP
Ctrl
E
r = 0.389
p = 0.0906
Spatial Eosinophil Score
Spatial Basal Immune Remodeling Score
F
Host defense
Immune remodeling
(Public data)
Cell Score
p < 2.22e-16
Ctrl
CRSsNP
eCRSwNP
G
WNT3A
WNT5B
SOX4
SOX9
KLF2
KLF6
Relative_expression
Pseudotime
Tissue type
CRSsNP_Eth
CRSwNP_NP
H
(Public data)
Running Enrichment Score
REACTOME_INTERLEUKIN_4_AND_INTERLEUKIN_13_SIGNALING
REACTOME_SIGNALING_BY_INTERLEUKINS
REACTOME_CELL_CELL_COMMUNICATION
REACTOME_CELL_JUNCTION_ORGANIZATION
pvalue
0.001458
0.0002511
0.0003029
I
Cell-fate signature enrichment in IL4/13 cytokine
stimulated Non-polyp derived basal cells
Immune remodeling
(p=0.01)
Host defense
Pre-branch
−log10(P-value)
High
Low
-log10(P-value)

FigureS5

A

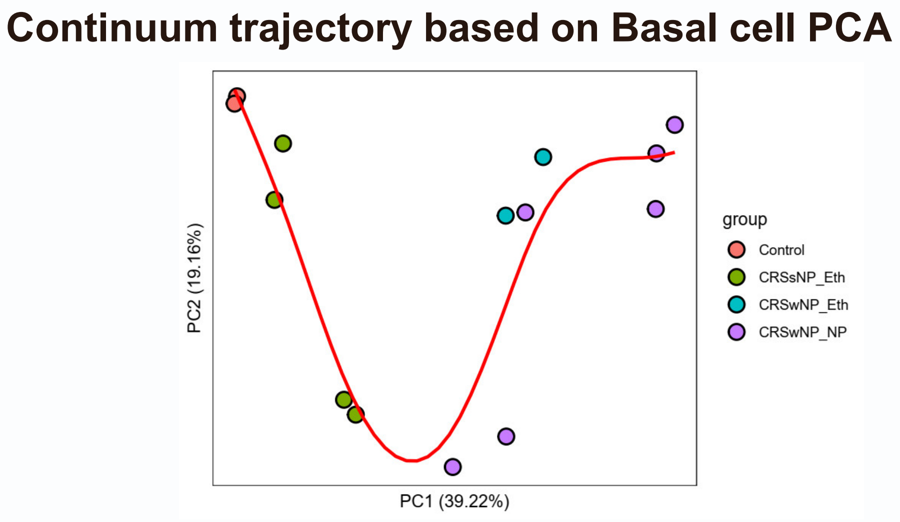
Continuum trajectory based on Basal cell PCA
PC2 (19.16%)
PC1 (39.22%)
group
Control
CRSsNP_Eth
CRSwNP_Eth
CRSwNP_NP

B

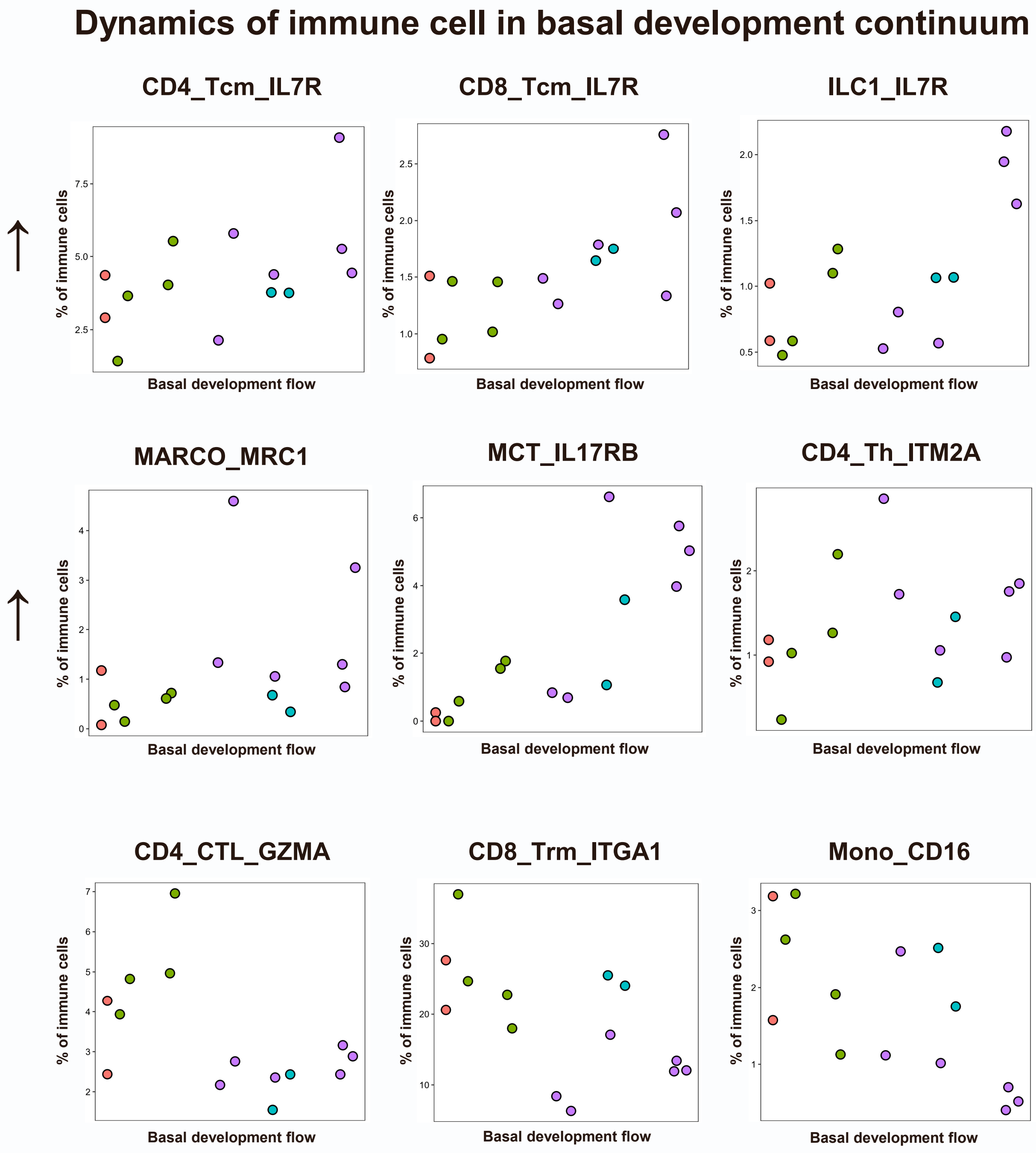
Dynamics of immune cell in basal development continuum
CD4_Tcm_IL7R
CD8_Tcm_IL7R
ILC1_IL7R
MARCO_MRC1
MCT_IL17RB
CD4_Th_ITM2A
CD4_CTL_GZMA
CD8_Trm_ITGA1
Mono_CD16
% of immune cells
Basal development flow

FigureS6

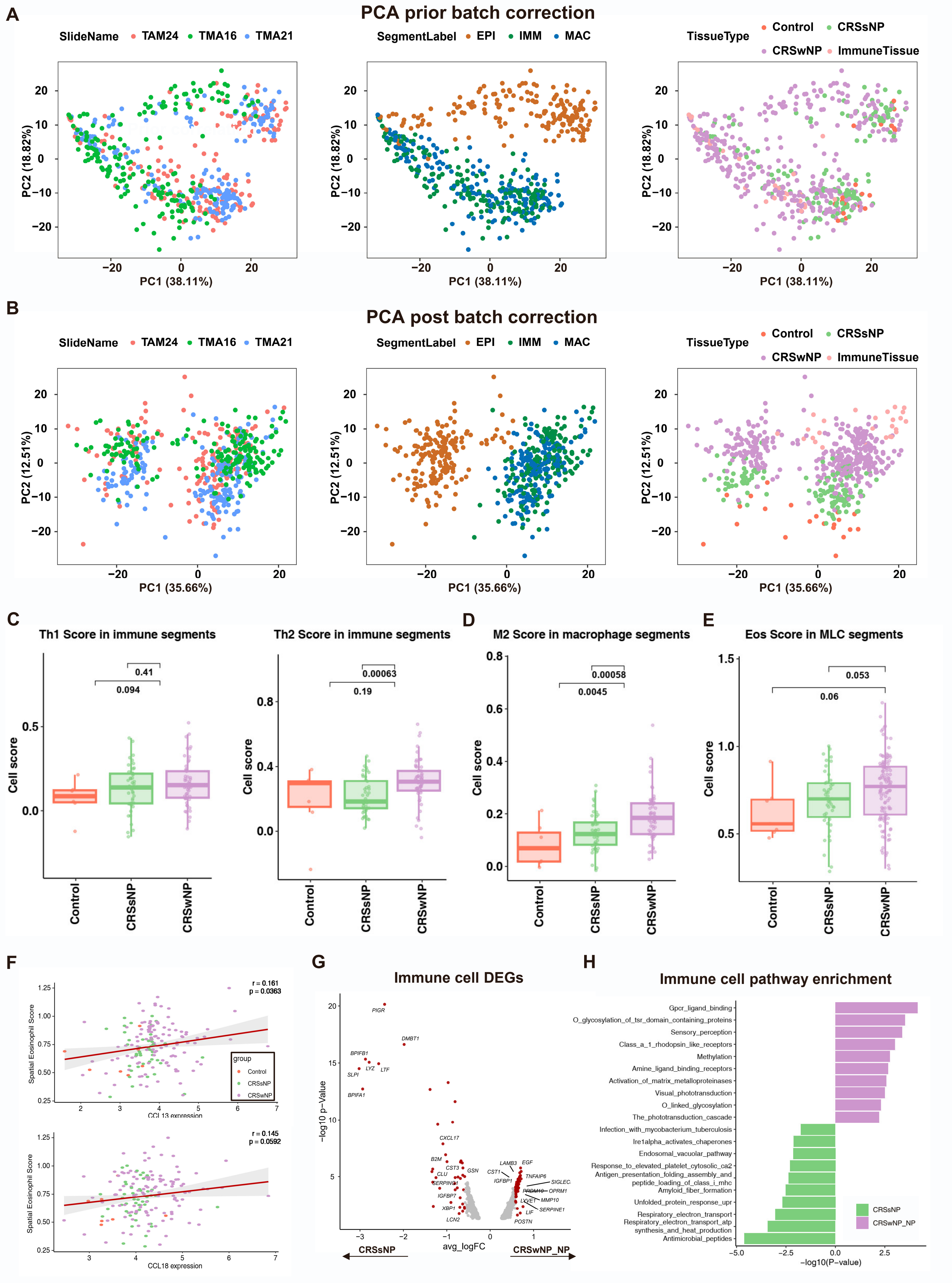

A
PCA prior batch correction
SlideName TAM24 TMA16 TMA21
SegmentLabel EPI IMM MAC
TissueType Control CRSsNP CRSwNP ImmuneTissue
PC2 (18.82%)
PC1 (38.11%)
B
PCA post batch correction
SlideName TAM24 TMA16 TMA21
SegmentLabel EPI IMM MAC
TissueType Control CRSsNP CRSwNP ImmuneTissue
PC2 (12.51%)
PC1 (35.66%)
C
Th1 Score in immune segments
0.41
0.094
Th2 Score in immune segments
0.00063
0.19
D
M2 Score in macrophage segments
0.00058
0.0045
E
Eos Score in MLC segments
0.053
0.06
Cell score
Control
CRSsNP
CRSwNP
F
r = 0.161
p = 0.0363
Spatial Eosinophil Score
CCL13 expression
group
Control
CRSsNP
CRSwNP
r = 0.145
p = 0.0592
Spatial Eosinophil Score
CCL18 expression
G
Immune cell DEGs
−log10 p−Value
avg_logFC
CRSsNP
CRSwNP_NP
PIGR
DMBT1
BPIFB1
LYZ
LTF
SLPI
BPIFA1
CXCL17
B2M
CLU
CST3
GSN
SERPING1
IGFBP7
XBP1
LCN2
LAMB3
EGF
CST1
IGFBP1
TNFAIP6
SIGLEC
PRDM10
OPRM1
LYVE1
MMP10
LIF
SERPINE1
POSTN
H
Immune cell pathway enrichment
Gpcr_ligand_binding
O_glycosylation_of_tsr_domain_containing_proteins
Sensory_perception
Class_a_1_rhodopsin_like_receptors
Methylation
Amine_ligand_binding_receptors
Activation_of_matrix_metalloproteinases
Visual_phototransduction
O_linked_glycosylation
The_phototransduction_cascade
Infection_with_mycobacterium_tuberculosis
Ire1alpha_activates_chaperones
Endosomal_vacuolar_pathway
Response_to_elevated_platelet_cytosolic_ca2
Antigen_presentation_folding_assembly_and peptide_loading_of_class_i_mhc
Amyloid_fiber_formation
Unfolded_protein_response_upr
Respiratory_electron_transport
Respiratory_electron_transport_atp synthesis_and_heat_production
Antimicrobial_peptides
CRSsNP
CRSwNP_NP
−log10(P−value)

Table.S3 Genesets used in cell scoring analysis, related to STAR Methods

Mφ Proinflammatory	Mφ Immunoregulatory	Th1	Th2
IL23	ARG1	IL2RA	IL4
TNF	ARG2	IL2RB	IL5
CXCL9	IL10	IL12B	IL10RA
CXCL10	CD32	IL12RB1	IL13
CXCL11	CD163	IL18	IL17RB
CD86	CD23	IL27	IL33
IL1A	CD200R1	STAT4	STAT5
IL1B	PDCD1LG2	TNF	STAT6
IL6	CD274	CXCL8	GATA3
CCL5	MARCO	CXCL9	MAF
IRF5	CSF1R	CXCL10	TSLP
IRF1	CD206	CXCL11	CCL13
CD40	IL1RN	CCL3	CCR4
IDO1	IL1R2	CCR5	
KYNU	IL4R		
CCR7	CCL4		
	CCL13		
	CCL20		
	CCL17		
	CCL18		
	CCL22		
	CCL24		
	LYVE1		
	VEGFA		
	VEGFB		
	VEGFC		
	VEGFD		
	EGF		
	CTSA		
	CTSB		
	CSTC		
	CTSD		
	TGFB1		
	TGFB2		
	TGFB3		
	MMP14		
	MMP19		
	MMP9		
	CLEC7A		
	WNT7B		
	FASL		
	TNFSF12		
	TNFSF8		
	CD276		
	VTCN1		
	MSR1		
	FN1		
	IRF4		

Eosinophil	Basal Cell-fate1	Basal Cell-fate2
ITGB2	SCGB1A1	CST1
ITGA4	WFDC2	GSN
ITGA6	TMEM213	KLF6
ITGAX	RARRES1	CLDN4
CD44	SERPINB3	SERPINB3
CD34	S100A4	KRT6A
SELL	LCN2	NOS2
CCR2	S100A9	CD55
CCR4	MT2A	ALOX15
CCR5	KRT8	CSTB
CCR6	FAM3D	CDH26
CCR7	IFI27	IGFBP5
CXCR4	SLPI	SERPINB4
IL2RA	AKR1C3	RAB11FIP1
IL3RA	MDK	NEAT1
IL4R	CLDN7	POSTN
IL5RA	TSPAN1	TXN
IL10RA	SERPINB4	SLC25A25
IL12RB1	KRT7	ANXA1
IL13RA1	AQP5	IGFBP3
IL17RA		
CSF2RB		
TGFBR1		
TGFBR2		
IFNGR1		
IFNGR2		
KIT		
TLR7		
NOD1		
NOD2		
AGER		
CR1		
C3AR1		
C5AR1		
